# Demonstration of a Direct Interaction between β_2_-Adrenergic Receptor and Insulin Receptor by BRET and Bioinformatics

**DOI:** 10.1371/journal.pone.0112664

**Published:** 2014-11-17

**Authors:** Maja Mandić, Luka Drinovec, Sanja Glisic, Nevena Veljkovic, Jane Nøhr, Milka Vrecl

**Affiliations:** 1 Institute of Anatomy, Histology & Embryology, Veterinary Faculty, University of Ljubljana, Ljubljana, Slovenia; 2 Development Department, Aerosol d.o.o., Ljubljana, Slovenia; 3 Center for Multidisciplinary Research, Institute of Nuclear Sciences VINCA, University of Belgrade, Belgrade, Serbia; 4 Department of Incretin & Islet Biology, Novo Nordisk A/S, Måløv, Denmark; Hungarian Academy of Sciences, Hungary

## Abstract

Glucose metabolism is under the cooperative regulation of both insulin receptor (IR) and β_2_-adrenergic receptor (β_2_AR), which represent the receptor tyrosine kinases (RTKs) and seven transmembrane receptors (7TMRs), respectively. Studies demonstrating cross-talk between these two receptors and their endogenous coexpression have suggested their possible interactions. To evaluate the effect of IR and prospective heteromerization on β_2_AR properties, we showed that IR coexpression had no effect on the ligand binding properties of β_2_AR; however, IR reduced β_2_AR surface expression and accelerated its internalization. Additionally, both receptors displayed a similar distribution pattern with a high degree of colocalization. To test the possible direct interaction between β_2_AR and IR, we employed quantitative BRET^2^ saturation and competition assays. Saturation assay data suggested constitutive β_2_AR and IR homo- and heteromerization. Calculated acceptor/donor (AD_50_) values as a measure of the relative affinity for homo- and heteromer formation differed among the heteromers that could not be explained by a simple dimer model. In heterologous competition assays, a transient increase in the BRET^2^ signal with a subsequent hyperbolical decrease was observed, suggesting higher-order heteromer formation. To complement the BRET^2^ data, we employed the informational spectrum method (ISM), a virtual spectroscopy method to investigate protein-protein interactions. Computational peptide scanning of β_2_AR and IR identified intracellular domains encompassing residues at the end of the 7^th^ TM domain and C-terminal tail of β_2_AR and a cytoplasmic part of the IR β chain as prospective interaction domains. ISM further suggested a high probability of heteromer formation and homodimers as basic units engaged in heteromerization. In summary, our data suggest direct interaction and higher-order β_2_AR:IR oligomer formation, likely comprising heteromers of homodimers.

## Introduction

The functional interplay between different classes of receptors represents a means of fine-tuning the control of cellular functions that could be fundamental for understanding pathologic conditions and responses to therapeutic agents that interact with cell-surface receptors. Originally, 7 transmembrane receptors (7TMRs) and receptor tyrosine kinases (RTKs), together with their respective downstream effectors, were thought to represent distinct and linear signaling units; however, recent studies have provided evidence for functional crosstalk between these different types of receptors [1].

Glucose metabolism is under the cooperative regulation of the RTK insulin receptor (IR) and β_2_-adrenergic receptor (β_2_AR), a representative 7TMR. The anabolic action of insulin promotes glycogen synthesis, glucose uptake in skeletal muscle and lipid storage. By contrast, catecholamines act in opposition to insulin, stimulating glycogen breakdown, gluconeogenesis, and lipolysis [2]. They play an important role in counter-regulation of insulin-induced hypoglycemia. The ability of insulin to counter regulate catecholamine action at the tissue/cellular level is crucial for the “tight” regulation of serum glucose levels, and interaction between IR and β_2_AR could be a required action in this process.

At the molecular level, the ability of insulin to counter regulate β_2_ARs can be exerted through insulin-stimulated phosphorylation of β_2_AR and its subsequent internalization. An early *in vitro* study demonstrated that, in the presence of insulin, IR catalyzes the phosphorylation of β_2_AR predominantly at residues located in the cytoplasmic tail of β_2_AR–i.e., Tyr350/Tyr354, and Tyr364 [3]–a finding that was consistent with the hypothesis that IR directly interacts with and phosphorylates β_2_AR. Phosphorylated β_2_AR Tyr350 creates a phosphotyrosine Src homology 2 (SH2) binding domain, which effectively precludes β_2_AR from coupling to its cognate G-protein (Gα_S_) and enables binding of the adaptor Grb2, the p85 regulatory subunit of phosphoinositide 3-kinase (PI3K) or the GTPase dynamin; all of the latter molecules are involved in 7TMR trafficking [4], [5], [6]. In addition, insulin-induced activation of MAPKs, ERK 1 and ERK 2, is potentiated by β_2_AR via a mechanism that requires the integrity of Tyr350 [4]. Thus, the functional cross-talk between β_2_AR and IR may be to fine tune the signals from multiple receptor signaling pathways. Recently, Fu et al. [7] described an IR:β_2_AR complex in the mouse heart, indicating that cross-talk between these two receptors could provide a molecular basis for the pathophysiology of metabolic and cardiovascular dysfunction under insulin-resistant states. β_2_AR and IR are also endogenously coexpressed in other cell types–i.e., pancreatic beta cells, adipocytes, liver cells, skeletal muscle cells and astrocytes [8], [9], [10]. Therefore, the formation of heteromeric complexes consisting of these two receptors may be plausible.

In the past decade, the concept that 7TMRs can exist as dimers or higher-order oligomers has advanced rapidly [11], [12], and biophysical techniques based on resonance energy transfer (RET) such as BRET have become methods of choice in receptor oligomerization studies (recently reviewed in [13], [14], [15], [16], [17], [18]). Utilizing BRET, β_2_AR homo- and heteromerization with different 7TMR family members have been reported [19], [20], [21], [22], [23], [24], [25].

In contrast to 7TMR-based BRET studies, BRET has been less exhaustively employed for RTK-based studies, and an overview of BRET studies associated with RTKs was recently reviewed by Issad et al. [26] and Siddiqui et al. [27]. RTKs often employ dimerization as a key factor in their activation; in fact, members of the IR subfamily are pre-dimerized by disulfide bonds (reviewed by De Meyts [28]). In IR studies, BRET was used to monitor the activation state of IR [29], [30], [31] and its interactions with different intracellular binding partners, including protein tyrosine phosphatase-1B [32], Grb14 [33] and IRS1, IRS4 and Shc [34]. IR can also form hybrid heterotetramers with the insulin-like growth factor-1 (IGF-1) receptor, exemplifying that IR can comprise heteromers that are products of independent genes [35].

In addition to interfamily receptor heteromerization–i.e., 7TMR:7TMR and RTK:RTK pairs–a physiologically relevant, direct physical interaction between the 7TMR adenosine A_2A_ receptor (A_2A_-AR) and RTK fibroblast growth factor receptor (FGFR) was demonstrated [36]. Evidence for other 7TMR:RTK complexes was also provided [37], [38], [39]. Computer-assisted analysis suggested that the N- and C-termini, as well as the third intracellular loop (ICL3) of 7TMRs show a propensity toward unstructured conformation, and could potentially interact with domains of other receptors/receptor-interacting proteins (reviewed in [40]).

To test whether β_2_AR and IR are prospective interaction partners, we employed the following methods: i) radioligand binding and enzyme-linked immunosorbent assay (ELISA) to determine the receptor-binding properties and surface expression, as well as to quantify receptor internalization processes; ii) confocal microscopy to detect possible colocalization between β_2_AR and IR; iii) BRET^2^ proximity assays consistent with the direct interaction between β_2_AR and IR; and iv) the informational spectrum method (ISM), a virtual spectroscopy method to investigate protein-protein interactions [41], [42], to identify important informational characteristics of the interaction between β_2_AR and IR and to predict domains potentially involved in their interaction. Prior studies have proved this bioinformatics approach to be effective in identifying protein–protein interaction partners and some of them were also confirmed experimentally [43], [44].

## Materials and Methods

### Materials

Molecular biology reagents, as well as the tissue culture media and reagents, were purchased from Sigma-Aldrich (St. Louis, MO, USA) and Gibco Invitrogen Corporation (Breda, the Netherlands), unless otherwise specified. [^125^I]-Iodopindolol was obtained from PerkinElmer (Boston, MA, USA). The ligands pindolol and isoproterenol were purchased from Sigma-Aldrich, and recombinant human insulin S100 was from Novo Nordisk A/S (Bagsværd, Denmark). Coelenterazine 400a was purchased from Biotrend Chemikalien GmbH (Köln, Germany). Anti-hemagglutinin (HA) high-affinity rat monoclonal antibodies were from Roche (Basel, Switzerland). Anti-rat horseradish peroxidase (HRP)-conjugated antibodies and anti-rat TRITC-conjugated antibodies were from Sigma-Aldrich.

### Receptor fusion constructs

Human HA-tagged β_2_AR (HA-β_2_AR) cDNA in the vector pcDNA3.1 was purchased from Missouri S&T cDNA Resource Center (University of Missouri-Rolla, USA). HA-β_2_AR C-terminally tagged with *Renilla luciferase* 8 (RLuc8) (β_2_AR-RLuc8) was made using standard molecular biology techniques and was verified by sequencing. WT human IR isoform A that lacks exon 11 and IR C-terminally tagged with the green fluorescent protein variant 2 (IR-GFP^2^) were generated and verified at Novo Nordisk A/S. C-terminally RLuc8-tagged IR (IR-RLuc8), C-terminally GFP^2^-tagged β_2_AR (β_2_AR-GFP^2^) and the membrane-inserted GFP^2^-tagged construct (GFP^2^-17aa) were the same as described previously [24], [34], [45]. All of the generated cDNA clones were inserted into the expression vector pcDNA3.1(+).

### Cell culture and transfection

HEK-293 cells (European Collection of Animal Cell Cultures, Salisbury, UK) were routinely maintained and passaged in Dulbecco’s modified Eagle’s Medium (DMEM) supplemented with 10% (v/v) heat-inactivated fetal calf serum, 2 mM Glutamax-I, penicillin (100 U/mL) and streptomycin (100 µg/mL) at 37°C in a humidified atmosphere of 5% (v/v) CO_2_. For transient transfection, HEK-293 cells were seeded at a density of ∼1×10^6^ cells per 60-mm tissue culture dish or at a density of 4×10^6^ cells per 75-cm^2^ flask and transfections were performed the following day using Lipofectamine 2000 according to the manufacturer’s instructions. Cells were harvested 48 h after transfection, the cell number was determined using a cover-slipped hemocytometer, and the cells were resuspended in Dulbecco’s PBS supplemented with Ca^2+^/Mg^2+^, 1 g/L glucose and 36 mg/L sodium pyruvate to a density of 1×10^6^ cells/mL, unless otherwise stated.

### Luminescence and fluorescence measurements

The expression levels of RLuc8- and GFP^2^-tagged receptor constructs were monitored by total luminescence and fluorescence measurements as previously described [46]. For luminescence measurements, ∼2×10^5^ of resuspended cells were distributed in 96-well microplates (white Optiplate; Packard BioScience, Meriden, CT, USA). After the addition of coelenterazine 400a to a final concentration of 5 µM, total luminescence was measured using a TriStar LB 942 microplate reader (Berthold Technologies, Bad Wildbad, Germany). For fluorescence measurements, ∼2×10^5^ of resuspended cells from the same transfections were plated in black 96-well FIA plates (Greiner Bio-One, Frickenhausen, Germany). Total fluorescence was measured using the TriStar LB 942 microplate reader with an excitation filter at 380 nm and an emission filter at 515 nm. Background values obtained with mock-transfected HEK-293 cells were subtracted in both measurements, and the mean values of triplicate wells/sample were then calculated.

### Receptor Binding Assay

To establish a relationship between the luminescence/fluorescence signals generated by RLuc8 and GFP^2^-tagged β_2_ARs and cell-surface receptor number, radioligand binding assays were carried out on whole cells as previously described [47]. After transfections with increasing amounts of cDNA (0.01 to 2 µg) for either β_2_AR-RLuc8 or β_2_AR-GFP^2^, cells were plated onto 24-well plates at a density of ∼1×10^5^ cells per well. An aliquot of cells (∼5×10^5^) from each transfection was also transferred into a 60-mm dish for total luminescence/fluorescence measurements as described above. For the whole-cell radioligand binding assay, cells were washed once with assay buffer (HEPES-modified DMEM with 0.1% bovine serum albumin (BSA)) before being incubated with the β_2_AR antagonist [^125^I]-iodopindolol (30,000 cpm/well) and increasing concentrations of unlabeled pindolol (10^–12^ to 10^−5 ^M final concentration) in assay buffer for 2 h at 4°C. Cells were then washed 3 times with ice-cold PBS and solubilized with 0.2 M NaOH and 1% sodium dodecyl sulfate (SDS) solution, and then the radioactivity levels were determined using a γ counter (LKB Wallac, Turku, Finland). Determination of the radioactivity levels was performed in triplicate. Binding parameters were determined from displacement curves generated by a sigmoidal dose-response curve fit (GraphPad Prism 5.0). Receptor density (B_max_), expressed as the receptor number per cell, was calculated as previously described by Ramsay et al. [48]. Whole-cell radioligand binding assays were also performed with cells expressing β_2_AR alone or in combination with IR. Displacement curves were generated using [^125^I]-iodopindolol (30,000 cpm/well) and increasing concentrations of isoproterenol or isoproterenol and insulin, and IC_50_ values were then determined using GraphPad Prism 5.0.

### ELISA

ELISA assays for the measurement of surface-expressed HA-β_2_AR and quantification of receptor internalization were performed as described previously [49]. Briefly, HEK-293 cells were transiently transfected with either β_2_AR-RLuc8 alone or β_2_AR-RLuc8 and IR-GFP^2^. One microgram of each receptor was used, and the total amount of cDNA used for transfection was kept uniform by adding empty pcDNA3.1 vector. After transfection, cells were seeded at a density of ∼1×10^5^ cells per well in a 24-well plate. An aliquot of cells (∼5×10^5^) from each transfection was also transferred into a 60-mm dish for total luminescence/fluorescence measurements as described above. After 48 h, cells were first subjected to a 2-h starvation period, and then were treated as required in HEPES-modified DMEM with 0.1% BSA for 30 min at 37°C before fixing with 4% paraformaldehyde for 20 min at 4°C. Cells were then washed 3 times in PBS and blocked (PBS containing 1% BSA) for 60 min at room temperature. Cells were kept at room temperature for all of the subsequent steps. First, cells were incubated for 2 h with anti-HA antibody at a 1∶600 dilution. After 3 washes, cells were incubated with anti-rat horseradish peroxidase-conjugated antibody at a 1∶1000 dilution. After extensive washing, the reaction was developed using the 3, 3′, 5, 5′-tetra-methylbenzidine (TMB) liquid substrate system. The enzymatic reaction was stopped after 30 min at 37°C by adding 0.2 N sulfuric acid. The absorbances were measured at 450 nm using the microplate reader Rosys Anthos 2010 (Anthos Labtec Instruments, Wals, Austria). Determinations were made in triplicate.

### Receptor internalization assay

The β_2_AR internalization assay was performed as described previously [47]. Briefly, HEK-293 cells were transiently transfected with either β_2_AR alone or β_2_AR and IR. One microgram of each receptor was used, and the total amount of cDNA used for transfection was kept uniform by adding empty pcDNA3.1 vector. After 2 days, cells were first subjected to a 2-h starvation period in assay medium (HEPES-modified DMEM with 0.01% BSA) before being incubated with 10 µM isoproterenol or a combination of 10 µM isoproterenol and 0.1 µM insulin S100 in assay medium for time intervals ranging from 5 min to 60 min at 37°C. Cells were then placed on ice, washed 3 times with ice-cold PBS and incubated for 2 h with [^125^I]-iodopindolol in the presence or absence of 10 µM pindolol at 4°C. Specific binding in each fraction was determined as the difference between radiolabeled ligand detected in the presence and absence of 10 µM pindolol. Receptor sequestration was then defined as the decrease in specific [^125^I]-iodopindolol binding compared with the total binding obtained in untreated cells. The amount of internalized receptors as a function of time was fitted using a one-site binding (hyperbola) curve fit (GraphPad Prism 5.0) to estimate the half-time of internalization (t_1/2_). All of the time points were performed in triplicate.

### Confocal microscopy

HEK-293 cells were transiently transfected with constructs encoding HA-tagged and/or GFP^2^-tagged receptor constructs, trypsinized, and plated on poly-L-lysine-coated glass coverslips in complete DMEM. After 48 h, cells were treated as required with either 10 µM isoproterenol, 0.1 µM insulin S100 or the combination of both in HEPES-modified DMEM for 10 min at 37°C. Upon treatment, cells were washed with ice-cold PBS and fixed with 4% paraformaldehyde for 20 min at 4°C. Following washing (3 times in PBS), cells were permeabilized with PBS containing 0.01% Triton X-100 for 20 min. To reduce the nonspecific binding, cells transfected with the HA-tagged receptors were incubated in blocking solution (PBS containing 1% BSA) for 30 min. Subsequently, cells were incubated with a 1∶100 dilution of primary rat anti-HA antibodies in PBS overnight at 4°C. Following washing (3 times in PBS), cells were incubated with a 1∶50 dilution of secondary rabbit anti-rat TRITC-conjugated antibodies for 60 min at room temperature in the dark. Cells were then mounted using an anti-fading ProLong Gold reagent (Molecular Probes, the Netherlands), sealed and examined under an oil immersion objective (Planapo 40×, N.A. = 1.25) using a Leica multispectral confocal laser microscope (Leica TCS NT, Heidelberg, Germany). The sequential detection of GFP^2^- and TRITC-stained receptors was achieved using excitation laser lines at 488 nm (argon) and 543 nm (helium-neon), respectively. The fluorescence from the channels was collected sequentially, and images were produced using an 8-fold frame averaging a resolution of 1024×1024 pixels. Optical sections (1.0 µm) were acquired, and representative sections corresponding to the middle of the cells were presented using Adobe Photoshop 7.0 computer software.

### BRET^2^ saturation and competition assays

To derive BRET^2^ saturation curves, HEK-293 cells were transiently cotransfected with constant amounts of the constructs encoding RLuc8-tagged receptors together with increasing amounts of the constructs encoding GFP^2^-tagged receptors. For BRET^2^ competition assays, HEK-293 cells were cotransfected with constant amounts of RLuc8- and GFP^2^-tagged receptor constructs and with increasing amounts of untagged receptors. BRET^2^ assays were performed as described previously [24], [46], [47]. Briefly, 180 µl of resuspended cells containing ∼2×10^5^ cells was distributed in 96-well microplates (white Optiplate; Packard BioScience, Meriden, CT, USA). After the addition of coelenterazine 400a to a final concentration of 5 µM using an injector, readings were collected (TriStar LB 942 microplate reader, Berthold Technologies, Bad Wildbad, Germany). Signals at 410 nm (RLuc8 luminescence signal) and 515 nm (emission of light from excited GFP^2^) were measured sequentially, and 515/410 ratios (BRET^2^ signal) were calculated. The results were expressed in milliBRET units (mBU); BRET ratio × 1000. The expression levels of RLuc8- and GFP^2^-tagged constructs for each experiment were assessed by total luminescence and fluorescence measurements as described above. Determinations were made in triplicates.

### BRET^2^ assay data evaluation

The BRET^2^ values were fitted using the following equation for dimers: BRET = BRET_max_(1/(1+AD_50_/X)), where X is the ratio of acceptor (A; Receptor-GFP^2^) to donor ((D; Receptor-RLuc8) molecules. BRET_max_ is the maximum BRET^2^ signal when all of the donor molecules are interacting with acceptor molecules. AD_50_ value corresponds to the acceptor/donor ratio providing 50% of the BRET_max_ and reflects the relative affinity of the acceptor (GFP^2^-tagged receptor) for the donor molecules (RLuc8-tagged receptor). Fitting parameters were compared using Welch’s t-test.

### Simplistic BRET model for trimers with different association affinities

The BRET model for trimers is based on the Veacht and Steyer (1977) concept [50]. The theoretical saturation curve for trimers with the same association affinity is:
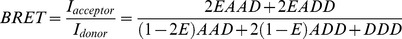
(1)where A, D and E are acceptor, donor and energy transfer efficiency, respectively. If we have different affinities for the formation of AAD trimer we introduce enhancement factor X:
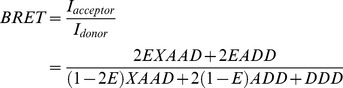
(2)


### BRET competition assay simulation for trimers with different energy transfer ratio

The BRET model for trimer competition is based on the Veacht and Steyer (1977) concept [50]. For receptors with equal affinities to form trimers we determine the frequency of each trimer type (A = acceptor, D = donor and W = wild type receptor):
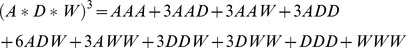
(3)


BRET is defined as a ratio of light emission from acceptor divided by that of the donor. E is resonance energy transfer ratio:
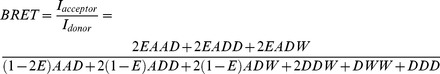
(4)


If the energy transfer ratio E is different for ADW trimers the following equation is obtained:
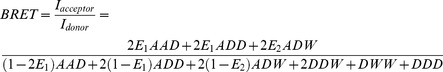
(5)


### Informational spectrum method (ISM)

The ISM is based on a model that assigns to each amino acid a defined parameter describing a physico-chemical property involved in the biological activity of the protein and corresponding to electron-ion interaction potential (EIIP). These values determine the electronic properties of the amino acids responsible for their intermolecular interactions [51].

The obtained numerical sequence, representing the primary structure of a protein, is then subjected to a discrete Fourier transformation defined as follows:
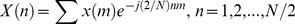
(6)where x(m) is the m-th member of a given numerical series, N is the total number of points in this series, and X(n) are discrete Fourier transformation coefficients. These coefficients describe the amplitude, phase and frequency of sinusoids, which comprised the original signal. The absolute value of the complex discrete Fourier transformation defines the amplitude spectrum and phase spectrum. The complete information concerning the original sequence is contained in both spectral functions. However, in the case of protein analysis, relevant information is presented in an energy density spectrum (for a review, see [51]) defined as follows:



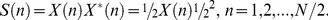
(7)


Thus, the initial information defined by the sequence of amino acids is now presented in the form of the informational spectrum (IS), representing the series of frequencies and their amplitudes.

The IS frequencies correspond to the distribution of structural motifs with defined physico-chemical characteristics responsible for the biological function of a protein. When comparing proteins that share the same biological or biochemical function, ISM allows the detection of code/frequency pairs that are specific for their common biological properties or correlate with their specific interaction. This common informational characteristic of sequences is determined by a cross-spectrum (CS) for two proteins or consensus informational spectrum (CIS) for two or more proteins–i.e., the Fourier transformation of the correlation function for the spectrum. In this way, any spectral component (frequency) not present in all of the compared ISs is eliminated. Peak frequencies in CIS are common frequency components for the analyzed sequences. A measure of similarity for each peak is the signal-to-noise ratio (S/N), representing the ratio between the signal intensity at one particular IS frequency and the main value of the whole spectrum. If a CIS is calculated for a group of proteins with different primary structures, and strictly defined peak frequencies are found, the analyzed proteins likely participate in a mutual interaction or have a common biological function. The ISM was, thus far, successfully applied in the structure-function analysis of different protein sequences [51], prediction of new protein interactors [43] and identification of protein domains responsible for long-range interactions [52], [53].

### Computational peptide scanning

Computational peptide scanning was used to define linear protein regions that contribute the most to the amplitude and signal to noise ratio at the characteristic frequency and, therefore, are responsible for the interaction(s) described by the particular spectral characteristic. To identify the regions with the highest amplitudes at predefined Fourier frequencies, the entire sequences of β_2_AR and IR were scanned by the ISM algorithm with overlapping windows of different lengths, leading to the identification of regions with the highest amplitudes at predefined Fourier frequencies.

### Datasets

The sequences used for bioinformatics analysis were retrieved from the Uniprot database with the following accession numbers: P07550 (human β_2_AR) and P06213 (human IR isoform A that lacks exon 11).

### Statistical analysis

Statistical significance was determined using Student’s t-test and Welch’s t-test. Differences were considered statistically significant at a *p* value less than 0.05.

### Ethics Statement

N/A.

## Results

### Characteristics of the β_2_AR fusion constructs

Pharmacological characterization of the human HA-tagged β_2_AR (HA-β_2_AR) fused at the C-terminus with either the energy donor RLuc8 or energy acceptor GFP^2^ was performed using radioligand binding assays (Table 1). The IC_50_ values of pindolol for HA-β_2_AR-RLuc8 and β_2_AR-GFP^2^ were in agreement with those obtained for HA-β_2_AR and were also in the range previously reported for β_2_AR-RLuc fusion construct [47]. IR fusion constructs were previously characterized [34]. Radioligand binding assays were also performed with HEK-293 cells cotransfected with the HA-β_2_AR and IR at a 1∶1 cDNA ratio. The obtained IC_50_ values for the tested β_2_AR ligands–i.e., pindolol and isoproterenol–were comparable in β_2_AR- and in β_2_AR- and IR-expressing cells. Similarly, heteromerization with β_3_AR did not affect the binding properties of β_2_AR [20]. Insulin neither competed for binding against [^125^I]-iodopindolol nor affected the IC_50_ values of pindolol (Table 2).

**Table 1:** Pharmacological properties of β_2_AR fusion constructs expressed in HEK-293 cells.

Receptor	IC_50_ (nM)
HA-β_2_AR	1.58±0.26
HA-β_2_AR-RLuc8	1.16±0.11
β_2_AR-GFP^2^	1.88±0.58
β_2_AR-RLuc	0.94±0.06^a^

a Data from Vrecl et al. [47].

HEK-293 cells expressing HA-β_2_AR, β_2_AR-GFP^2^ or HA-β_2_AR-RLuc8 were incubated with [^125^I]-iodopindolol and increasing concentrations of pindolol (10–^12^ to 10–^5^ M final concentration). IC_50_ values were generated using a sigmoidal dose-response curve fit (GraphPad Prism 5.0). Data are expressed as the means ± S.E. of three independent experiments performed in triplicate.

**Table 2:** Effect of IR coexpression on the pharmacological properties of β_2_AR in HEK-293 cells.

	IC_50_ (nM)	
Ligands	HA-β_2_AR	HA-β_2_AR+IR
pindolol	1.58±0.26	1.15±0.15
isoproterenol	576.7±134.2	406.5±36.5
insulin	ND	ND
pindolol+insulin	2.73±0.58	1.13±0.23

ND – not detected.

HEK-293 cells expressing HA-β_2_AR alone or HA-β_2_AR together with IR at a 1∶1 cDNA ratio were incubated with [^125^I]-iodopindolol and increasing concentrations of the indicated ligands (10–^12^ to 10–^5^ M final concentration). When the concomitant effect of pindolol and insulin was tested, insulin was added to a 0.1 µM final concentration. IC_50_ values were generated using a sigmoidal dose-response curve fit (GraphPad Prism 5.0). Data are expressed as the means ± S.E. of three independent experiments performed in triplicate.

### β_2_AR surface expression and the internalization effect of IR coexpression

**Figure 1:**
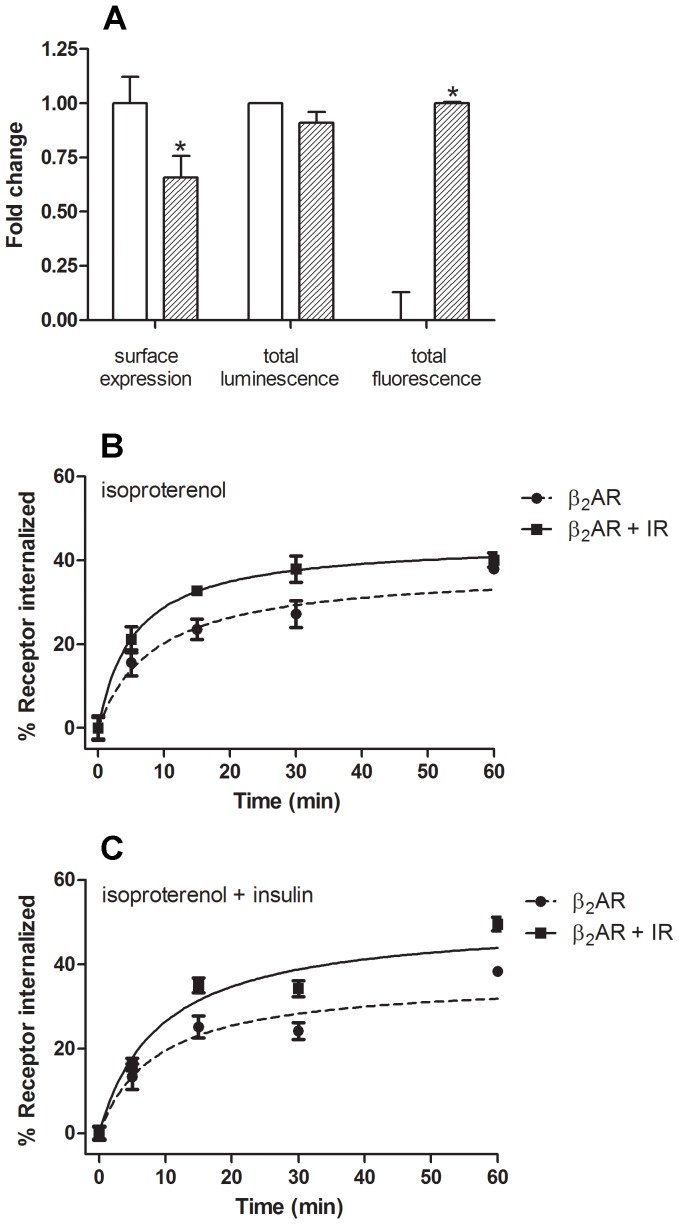
Effect of IR coexpression **on β_2_AR** surface expression and internalization in HEK-293 cells. **(A)** ELISA was performed on intact HEK-293 cells transiently transfected with HA-β_2_AR-RLuc8 alone (open bars) or HA-β_2_AR-RLuc8 together with IR-GFP^2^ (hatched bars) at a 1∶1 cDNA ratio using an antibody directed against the HA epitope. Antibody binding, as an index of receptor surface expression, was determined by measuring the absorbance at 450 nm. HA-β_2_AR-RLuc8 and IR-GFP^2^ total relative expression was determined by luminescence and fluorescence measurement, respectively. Total luminescence was measured after the addition of the RLuc substrate coelenterazine 400a. Total fluorescence was measured with an excitation filter at 380 nm and an emission filter at 515 nm. Fold change is relative to open bars for surface expression and total luminescence, and relative to hatched bars for total fluorescence. Data are expressed as the means ± S.E. of three independent experiments performed in triplicate. *, p<0.05 as compared with HA-β_2_AR-RLuc8 transfected cells. **(B, C)** Effect of IR coexpression on the time-course of β_2_AR internalization. HEK-293 cells were transiently transfected with either β_2_AR (dotted line) or β_2_AR together with the IR (solid line) at a 1∶1 cDNA ratio. β_2_AR internalization was first induced by (B) isoproterenol (10 µM) or (C) the combination of isoproterenol (10 µM) and insulin (0.1 µM) for the indicated time intervals. Receptor sequestration was then defined as the decrease in specific [^125^I]-iodopindolol binding compared with the total binding obtained in untreated cells. The amount of internalized receptors as a function of time was fitted using a one-site binding (hyperbola) curve fit (GraphPad Prism 5.0). Data are expressed as the means ± S.E. from three independent experiments performed in triplicate.

The effect of IR coexpression on β_2_AR surface expression and internalization properties was evaluated by ELISA in intact cells and by radioligand binding assays. As shown in Fig. 1A, a significant approximately 35% decrease in β_2_AR surface expression was observed in HEK-293 cells cotransfected with IR compared with β_2_AR-expressing cells (p<0.05). However, total β_2_AR expression was not affected by IR coexpression as shown by luminescence measurements, whereas IR expression was validated by fluorescence measurements. Analogous observations in β_2_AR surface expression were detected using radioligand binding assays, where β_2_AR surface expression decreased by around 30% (27.89±2.11) in HEK-293 cells cotransfected with IR compared with β_2_AR-expressing cells (p<0.05).

**Figure 2:**
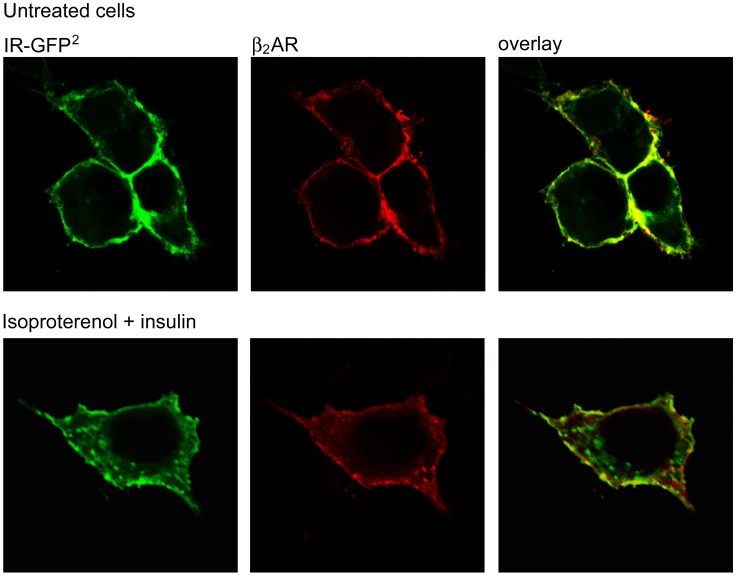
Visualization of β_2_AR and IR cellular localization by confocal microscopy. IR-GFP^2^ and HA-β_2_AR-RLuc8 cellular localization is shown in untreated (control) cells (upper panels) and cells concomitantly treated with isoproterenol (10 µM) and insulin (0.1 µM) for 10 min at 37°C (lower panels). The green color indicates IR-GFP^2^; red indicates HA-β_2_AR-RLuc8; yellow/orange is the overlapping region indicating colocalization of IR-GFP^2^ and HA-β_2_AR-RLuc8. Note that both receptors have comparable localization in untreated and agonist-stimulated cells and that they exhibit a high degree of colocalization. However, in agonist-stimulated cells a proportion of intracellular receptors did not colocalize. Objective 40× and zoom factor 4 apply for all images.

Additionally, ELISA measurements suggested that IR coexpression moderately increased isoproterenol-induced β_2_AR internalization, whereas concomitant treatment with isoproterenol and insulin evoked a comparable internalization rate. Treatment with insulin did not induce marked β_2_AR internalization (Figure S1). To further examine the internalization kinetics of β_2_AR, HEK-293 cells expressing either β_2_AR or β_2_AR and IR were treated with either isoproterenol (10 µM) or a combination of both isoproterenol (10 µM) and insulin (0.1 µM) for varying periods of time (5 min to 1 h), and the number of cell surface binding sites were determined (Fig. 1B and C). We found that isoproterenol-induced steady-state proportion of internalized receptors was approximately 38% and 44% in β_2_AR- and in β_2_AR- and IR-expressing cells, respectively. The rate of isoproterenol-induced β_2_AR internalization was significantly increased by coexpression of IR (p<0.05), as the estimated t_1/2_ was 8.6±1.3 and 5.4±0.6 min in β_2_AR- and in β_2_AR- and IR-expressing cells. Comparable results were obtained when either β_2_AR- or β_2_AR- and IR-expressing cells were concomitantly treated with isoproterenol and insulin (see Fig. 1B and C). In addition, confocal fluorescence microscopy demonstrated predominant plasma membrane localization of the HA-β_2_AR-RLuc8 and IR-GFP^2^ and their colocalization in untreated cells (Fig. 2, upper panels; untreated cells). Concomitant treatment of β_2_AR- and IR-expressing cells with isoproterenol and insulin (Fig. 2, lower panels) promoted receptor redistribution to the cytoplasm, indicating receptor internalization. Again, agonist-redistributed β_2_AR and IR showed a similar distribution pattern; however, a proportion of intracellular receptors did not colocalize.

### Interaction between the β_2_AR and IR–BRET evidence

Considering the observed effects of IR coexpression on β_2_AR surface expression and internalization, as well as colocalization of both receptors in HEK-293 cells, we next investigated whether proximity indicative of direct interaction occurs between β_2_AR and IR by performing BRET dilution, saturation and competition assays. To obtain an absolute value of donor [D] and acceptor [A] molecules–i.e., Receptor-RLuc8 and Receptor-GFP^2^–we first generated correlation curves between total luminescence and fluorescence *versus* the number of receptor-binding sites determined by radioligand binding. The linear correlation was obtained between the number of HA-β_2_AR-RLuc8 and β_2_AR-GFP^2^ binding sites and the total luminescence and total fluorescence, respectively (Figure S2).

The derived correlation factors to convert total luminescence and total fluorescence into receptor number were 0.00031 and 0.0089, respectively. Thereafter, BRET^2^ dilution assays with a constant [*A*]/[*D*] ratio were performed to set the concentration range for the saturation assays and distinguish monomers from dimers as previously described [54]. Due to the increasing noise in calculated BRET at low luminescence intensities, the lowest amount of HA-β_2_AR-RLuc8 and IR-RLuc8 cDNA used for transfection in saturation assays was determined at 0.05 and 0.015 µg, respectively.

**Figure 3:**
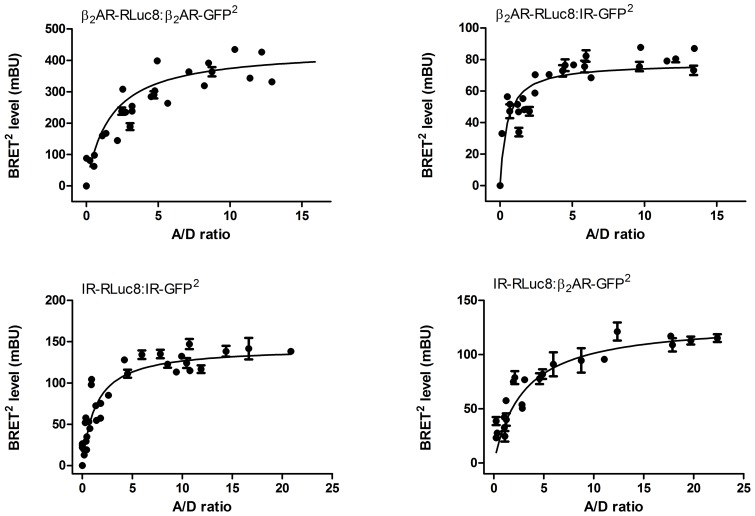
BRET^2^ saturation curves of β_2_AR and IR homo- and heteromers. HEK-293 cells were transiently cotransfected with a constant amount of RLuc8-tagged and increasing amounts of GFP^2^-tagged receptor encoding constructs. BRET^2^ values were plotted as a function of the ratio between the acceptor and donor fusion proteins (A/D ratio). A dataset is composed of 3–5 independent saturation experiments that were fitted using a nonlinear regression equation assuming a single binding site using GraphPad Prism 5.0 (Fitting results are found in Table 3).

BRET^2^ saturation assays were performed to investigate homo- and hetero-merization of β_2_AR and IR. The BRET^2^ signal was measured in live HEK-293 cells that were transiently transfected with constant amounts of RLuc8-tagged (HA-β_2_AR-RLuc8, IR-RLuc8) and with increasing amounts of GFP^2^-tagged (β_2_AR-GFP^2^, IR-GFP^2^) receptor encoding constructs. In dimers and higher oligomers, the probability of BRET interaction increases with increasing acceptor/donor ratio until all of the acceptors have pairs, and the maximum BRET^2^ level (BRET_max_) or plateau is reached. The AD_50_ value (also designated as BRET_50_; see methods) was also included to compare the affinity for homo−/heteromer formation. BRET^2^ saturation experiments data are shown in Fig. 3 and the expression levels of RLuc8- and GFP^2^-tagged receptor constructs are shown in Figure S3. For all of the receptor combinations, the BRET^2^ signal was plotted as a function of the A/D ratio increased as a hyperbolic function reaching a saturation level (Fig. 3). Control experiments for the specificity of the interaction were performed by cotransfecting RLuc8-tagged receptors with increasing amounts of membrane-inserted GFP^2^-tagged construct (GFP^2^-17aa). Plasma membrane localization of the GFP^2^-17aa construct was previously demonstrated [45]. The BRET^2^ signal increased linearly with the increase in fluorescence/luminescence (GFP^2^/RLuc8) ratio, most likely reflecting random collisions between the RLuc8-tagged receptors and control unrelated GFP^2^-tagged construct (GFP^2^-17aa) (Figure S4). Saturation assay data were fitted using a dimer model in the approximation of small energy transfer [54] (Table 3). We observed the highest BRET_max_–i.e., 437 mBU in the β_2_AR homologous saturation assay and considerably lower BRET_max_ values for other receptor combinations. It should be stressed that the BRET_max_ value depends on the distance between BRET pairs and is not a measure of the strength of interaction between the acceptor- and donor-tagged receptors. In homologous saturation assays, the value of AD_50_ for the IR homomer was significantly lower (p≤0.05) than that for the β_2_AR homomer (Table 3). For the receptor complex consisting of β_2_AR-RLuc8:IR-GFP^2^, the AD_50_ value was significantly lower (p≤0.05) than the AD_50_ value for the IR-RLuc8:β_2_AR-GFP^2^. For heterodimers, there should be no difference in the relative affinity between AD and DA dimers. To interpret our data, we developed a simplistic trimer model for interpretation of AD_50_ values in a BRET saturation assay. According to this model, different AD_50_ values are derived from theoretical BRET saturation curves with different affinities for various types of trimer formation (Figure S5). This model should be used with caution because it does not take into account the simultaneous formation of dimers, trimers and higher-order oligomers. Stimulation with the agonists insulin or isoproterenol did not promote any detectable change in the BRET^2^ signal (data not shown), indicating that the receptor dimers/oligomers form constitutively and that addition of agonists does not induce a detectable change in the conformational or oligomerization state of the receptor complexes. However, it needs to be stressed that BRET only provides information concerning a steady-state population of dimer/higher-order oligomers and is not suited to monitor the rapid, “real-time” dynamic type of di−/oligomerization [55].

**Table 3:** BRET^2^ saturation assay fitting results.

Receptor pair	BRET_max_ (mBU)	AD_50_
**β_2_AR-RLuc8:β_2_AR-GFP^2^**	437±35	1.7±0.5
**IR-RLuc8:IR-GFP^2^**	143±10	1.3±0.4
**β_2_AR-RLuc8:IR-GFP^2^**	78±4	0.5±0.1
**IR-RLuc8:β_2_AR-GFP^2^**	131±11	2.9±0.7

BRET^2^ data from saturation assays were fitted using the following equation for dimers: BRET = BRET_max_(1/(1+AD_50_/X)) where X is the ratio of acceptor (A; Receptor-GFP^2^) to donor (D; Receptor-RLuc8) molecules. The BRET_max_ is the maximal BRET obtained for a given pair and AD_50_ value corresponds to the A/D ratio providing 50% of the BRET_max_. The best-fit parameters and standard errors were derived from the data presented in Fig. 3. Fitting parameters were compared using Welch’s t-test. Statistical analysis shows that AD_50_ and BRET_max_ values differ significantly (p≤0.05) between all of the tests.

**Figure 4:**
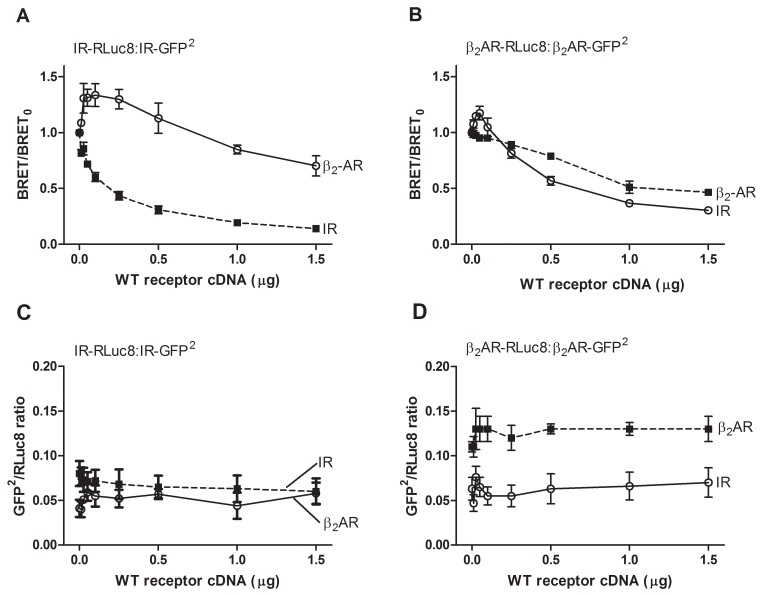
Homologous and heterologous BRET^2^ competition assay. (A, B) HEK-293 cells were cotransfected with a constant amount of RLuc8- and GFP^2^-tagged receptor while increasing the amount of untagged receptor. In the homologous competition assay (dotted line), BRET^2^ signals decreased with an increasing amount of WT receptor confirming the competition effect. In the heterologous BRET^2^ assay (solid line), where different WT receptors were used to compete with the tagged homomer receptor pair, a transient increase in the BRET^2^ signal with a subsequent hyperbolical decrease was observed. BRET_0_ is the BRET^2^ signal obtained in the absence of competitor. Data are expressed as the means ± S.E. from three independent experiments performed in triplicate. (C, D) Receptor-GFP^2^/Receptor-RLuc8 expression ratio (GFP^2^/RLuc8 ratio) in each sample was evaluated for total luminescence and total fluorescence. Total luminescence and total fluorescence was measured as described under Material and Methods. Note that GFP^2^/RLuc8 ratio was roughly constant in the absence or presence of increasing concentrations of competitor (untagged β_2_AR or IR). Data are expressed as the means ± S.E. from three independent experiments performed in triplicate.

To further support the findings from the saturation assays, we performed homologous and heterologous BRET^2^ competition assays where HEK-293 cells were co-transfected with a constant amount of RLuc8- and GFP^2^-tagged receptor while increasing the amount of untagged receptor (Fig. 4A and B). Competition experiments were carried out at the constant Receptor-GFP^2^/Receptor-RLuc8 expression ratio to avoid possible variations in the BRET^2^ signal due to fluctuation in the relative expression levels of the energy donor and acceptor (Fig. 4C and D). It is expected that the BRET^2^ signal would decrease if untagged receptors compete with the tagged receptors for the binding in complexes. In homologous BRET^2^ competition assays BRET^2^ signal decreased with increasing amount of competitor (un-tagged receptor). In the IR homologous competition assay, we observed a typical competition curve for dimers, where introduction of the same amount of untagged receptor produced an approximately 50% reduction in the observed BRET signal. In the β_2_AR homologous competition assay, the reduction of the BRET signal was smaller. This observation cannot be explained by a simple dimer or trimer model; it could be attributed to clustering of the β_2_AR where several acceptors can interact with each donor. In heterologous competition assays, we observed a transient increase in the BRET signal with a later hyperbolical decrease (see Fig. 4A and B). Untagged β_2_AR caused approximately 1.4-fold increase in the IR BRET signal, while the effect of untagged IR on the β_2_AR BRET signal was less obvious; maximal observed increase was less than 1.2-fold. The transient increase in the BRET signal with a subsequent hyperbolical decrease is theoretically predicted for trimers or higher-order oligomers, where the donor, acceptor and competitor are all present in the same complex (Figure S6).

### Interaction between β_2_AR and IR characterized by ISM

**Figure 5:**
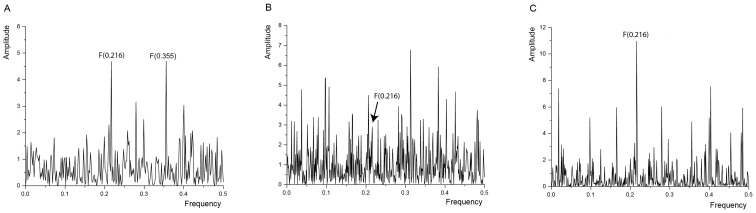
Informational spectrum (IS) of (A) β_2_AR, (B) IR and (C) cross-spectrum (CS) of β_2_AR and IR. CS of β_2_AR and IR revealed common information corresponding to the IS frequency F(0.216) (panel C).

To support our experimental evidence with the bioinformatics data, we next applied the informational spectrum method (ISM), a virtual spectroscopy method to investigate protein-protein interactions and to analyze the structure/function relationship of proteins. ISM was utilized to identify important informational characteristic of the interaction between β_2_AR and IR and identify the structural determinants potentially involved in receptor heteromerization. The primary structure of proteins encodes the information represented by the informational spectrum (IS) frequencies that correspond to the protein biological function. Mutually interacting proteins share common information that is represented by peaks in their cross-spectrum [51]. The informational spectrum of β_2_AR is presented in Fig. 5A. It contains two characteristic peaks at the frequency F(0.216) and F(0.355). Fig. 5B represents the IS of the IR. By performing cross-spectral analysis of β_2_AR and IR, we have identified that these two molecules share common information corresponding to the IS frequency F(0.216) (Fig. 5C). To further evaluate the importance of the peak at F(0.216) we performed CS analysis of β_2_AR and IR with scrambled IR and β_2_AR proteins as negative controls. The scrambled proteins with the identical amino acid composition to that of β_2_AR and IR were created by random permutation of original proteins. CS analysis between wild type receptors and randomly selected scrambled β_2_AR and IR are presented in Figure S7. The intensity of whole spectrum and the value of amplitudes at the characteristic peak F(0.216) is higher in CS of two wild type proteins compared to CS of original and scrambled proteins confirming the importance of the characteristic peak at the F(0.216) for interaction between β_2_AR and IR.

### Identification of the key protein domains responsible for the interaction between β_2_AR and IR

**Figure 6:**
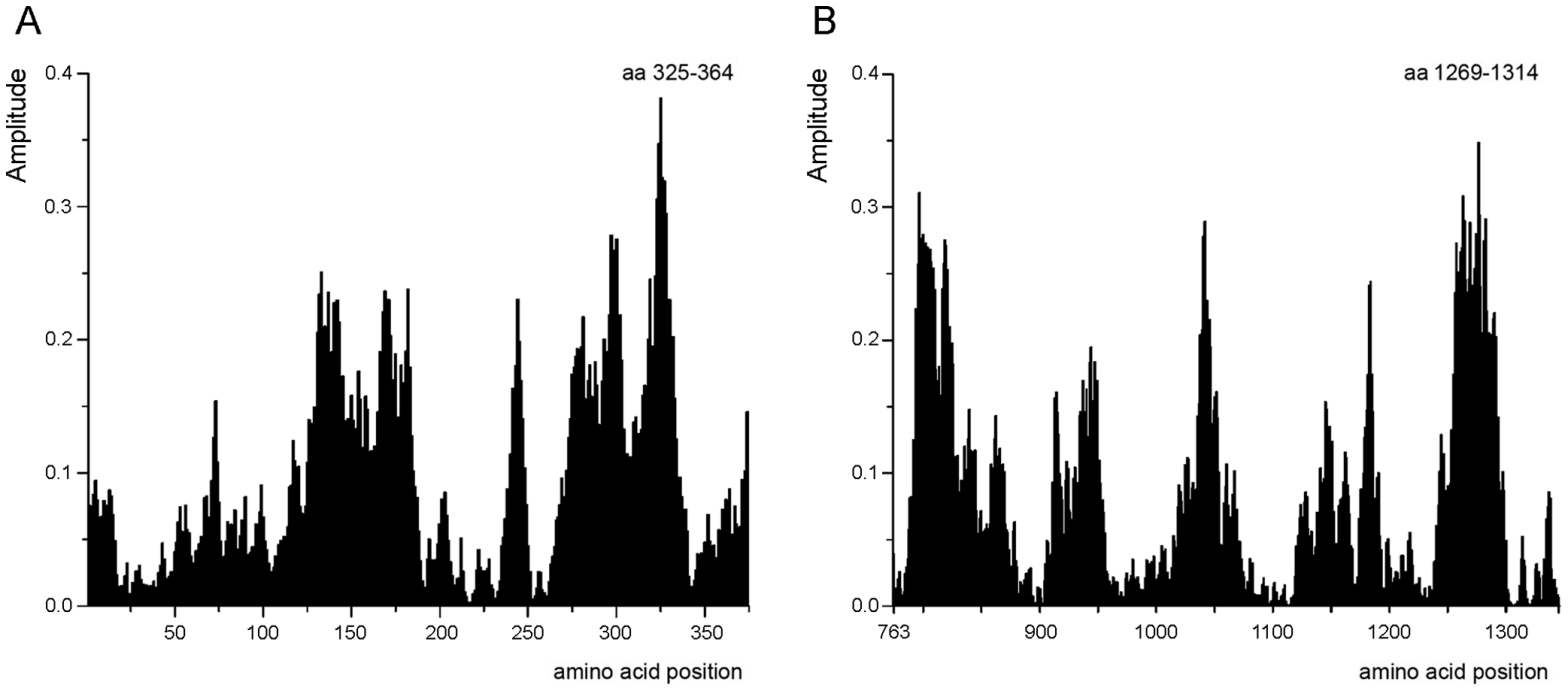
Mapping of the domains with maximal contribution to the frequency component F(0.216) in the informational spectrum of (A) β_2_AR and (B) IR. Peptide scanning of the β_2_AR and IR identified regions encompassing residues at the end of the 7^th^ TM domain and C-terminal tail of β_2_AR and a cytoplasmic part of the IR β chain as prospective interaction domains. The regions encompassing residues 325–364 in β_2_AR (A) and 1269–1314 IR (B) are essential for the information represented by the frequency F(0.216). The position of the first amino acid (aa) in the domain is shown. Panel B; the amino acid position denote the positions in IR β subunit starting from amino acid 763.

Computational peptide scanning of β_2_AR and IR was performed to identify the regions of proteins essential for information corresponding to the frequency F(0.216). The computer-assisted peptide scanning survey of the primary structure of β_2_AR with overlapping windows of different lengths revealed that the region encompassing residues 325–364 is essential for the information represented by the frequency F(0.216) (Fig. 6A). Further peptide scanning of IR identified three principal regions as important for the information represented by the frequency F(0.216), however region encompassing residues 1269–1314 represents the most probable domain involved in this interaction (Fig. 6B).

### Affinity of interaction between protomers

Peak frequencies in CIS represent common information encoded by the primary structures of analyzed proteins. Significance of information is determined by the signal-to-noise ratio (S/N), representing the ratio between the signal intensity at one particular IS frequency and main value of the whole spectrum. A higher S/N value at the characteristic frequency (F) in CS/CIS of two or more proteins suggests a higher propensity for their interaction.

**Figure 7:**
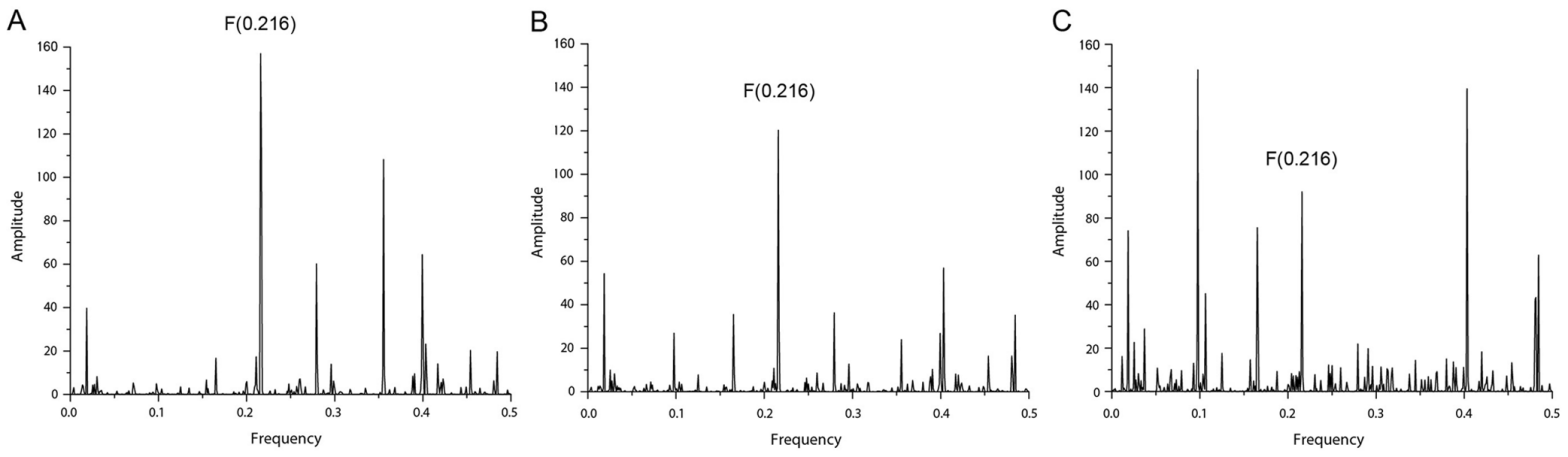
Consensus informational spectrum (CIS) of β_2_AR:IR tetramers with the characteristic peak at F(0.216). (A) β_2_AR:β_2_AR:β_2_AR:IR, (B) β_2_AR:β_2_AR:IR:IR and (C) β_2_AR:IR:IR:IR tetramers. Note that the interaction affinities among the receptors decreased in the order β_2_AR:β_2_AR:β_2_AR:IR≥β_2_AR:β_2_AR:IR:IR>β_2_AR:IR:IR:IR.

The current analysis showed that the interaction affinities between the homomers of β_2_AR and IR are similar and that at the level of dimer formation, both β_2_AR and IR displayed a higher propensity toward homodimerization than heterodimerization (Table 4). Considering that in the CS of the β_2_AR and IR, the two receptors share common information corresponding to the IS frequency F(0.216), it can be assumed that this frequency is equally important for heterodimerization and for higher-order hetero-oligomer formation. The obtained S/N values at F(0.216) in the CS of β_2_AR:β_2_AR:β_2_AR:IR and β_2_AR:β_2_AR:IR:IR tetramers were comparable, whereas that for the IR trimer displayed a considerably lower affinity for interaction with the β_2_AR monomer (Fig. 7 and Table 4).

**Table 4:** The affinity of interaction between the β_2_AR and IR homo- and heteromers characterized by the signal-to-noise ratio (S/N) at the characteristic frequency (F) in the consensus informational spectrum (CIS).

Dimer	S/N ratio
β_2_AR:β_2_AR	24.082
β_2_AR:IR	15.951
IR:IR	22.407
**Trimer**	
β_2_AR:β_2_AR:β_2_AR	52.867
β_2_AR: β_2_2AR:IR	43.476
β_2_AR:IR:IR	25.605
IR:IR:IR	51.146
**Tetramer**	
β_2_AR:β_2_AR:β_2_AR:β_2_AR	81.703
β_2_AR:β_2_AR:β_2_AR:IR	76.505
β_2_AR:β_2_AR:IR:IR	71.105
β_2_AR:IR:IR:IR	29.468
IR:IR:IR:IR	89.617

## Discussion

7TMRs form the largest and most important pharmacotherapeutic target in drug discovery. Continual discovery of receptor heteromers expands the repertoire of functional 7TMR units and offers promising new targets for drug development (reviewed in [56]). Increasing evidence for the existence of functional 7TMR:RTK heteromers [7], [36], [37], [38], [39] adds another level of complexity to this system. In the present study, we combined *in vitro* experimental and bioinformatics approaches for the investigation of the interaction between β_2_AR and IR. Initially, it was observed that IR coexpression reduces β_2_AR surface expression and accelerates its internalization. It could be assumed that IR increased constitutive internalization of the β_2_AR [57], thereby reducing its surface expression. The extent of isoproterenol-induced β_2_AR internalization was comparable to the previously reported internalization rate for β_2_AR [58], [59]. The estimated t_1/2_ of ∼9 min for isoproterenol-induced β_2_AR internalization is also within the range reported for the same receptor using a fluorimetric assay [60]. Augmented agonist-induced β_2_AR internalization promoted by IR coexpression is in good agreement with that described in a previous report [61]. The amount of surface-expressed β_2_AR lost to internalization in response to insulin in HEK-293, ∼15%, correlated well with results obtained in DT_1_MF-2 smooth muscle cells and CHO cells [61], [62] but was smaller than the reported value of ∼30% in epidermoid carcinoma A431 cells [6]. Our colocalization results in untreated cells correlate well with recent coimmunoprecipitation findings demonstrating that β_2_AR and IR form complexes in the mouse heart, in which the stimulation of IR resulted in the reduction of the association of the β_2_AR:IR complex [7].

BRET results provided further evidence for the direct interaction between β_2_AR and IR. Hyperbolic saturation assay curves are indicative of a specific constitutive interaction between IR and β_2_AR. The BRET_max_ obtained for β_2_AR homomerization is higher than that described in previous reports [19], [23], [24], whereas the relative affinity of β_2_AR for homomerization is within the previously reported range (AD_50_ of 1.7±0.5 *vs.* 1.2 ± 0.33 [23]). The RLuc8/GFP^2^ BRET^2^ donor/acceptor pair used in our study has the largest Förster distance *R_0_*_,_ leading to 50% of energy transfer from the donor to acceptor, thereby extending the maximum distance that the BRET^2^ system can accurately measure [63]. Lower reported BRET_max_ values were obtained with original BRET^1^ [19] or BRET^2^ [23], [24] combinations. The BRET_max_ values generated in the homologous IR saturation assay were also slightly higher than that previously reported [29]. The same study revealed that the substantial insulin-induced effect on the BRET signal could only be detected with partially purified fusion IRs [29]. Single-molecule analysis of fluorescently labeled 7TMRs recently demonstrated that β_2_AR had a high tendency of forming dimers, even at low densities comparable to receptor expression in native tissue; however, at higher densities, β_2_AR formed a mixture of di-, tri-, and tetramers [55]. Membrane-driven spatial organization of β_2_AR also favors its stable/extensive oligomerization [64]. The inability of agonists to modify the oligomerization status of β_2_AR was also reported in studies that utilized methods other than BRET (reviewed by Ferre et al. [56]). Both receptors displayed a similar propensity to form homomers as assessed by AD_50_ and S/N values. Affinities for the formation of β_2_AR:IR and IR:β_2_AR heteromers differed substantially, a finding that could not be explained by a dimer model that assumed a heteromer stoichiometry of 1∶1. The lower AD_50_ value observed for the β_2_AR-RLuc8:IR-GFP^2^ pair could suggest either a higher affinity for heteromer formation or, alternatively, complex oligomer formation. The melatonin MT_2_ receptor also displayed a higher propensity for heteromerization with the MT_1_ receptor [65], whereas certain receptor pairs (i.e., β_1_AR:β_2_AR [23], [24], β_2_AR:β_3_AR [20]), oxytocin and vasopressin receptors [66] showed a similar propensity, and muscarinic acetylcholine receptors [67] showed a slightly lower propensity for heteromerization. However, simplistic model for interpretation of AD_50_ values and heterologous competition assays data provided evidence for higher-order oligomeric complex formation. A transient increase in the BRET signal suggests that the energy transfer efficiency E is increased in hetero-oligomeric complexes due to the smaller distance between the BRET donor/acceptor pair in the complex (see Figure S6). A similar effect–i.e., an increased homo-dimer BRET signal induced by an unrelated, untagged receptor–was previously observed with the gastric inhibitory polypeptide receptor [24]. Therefore, both the change in affinities and transient increase in the BRET signal could be a reflection of higher-order oligomeric complexes with affinities for distinct associations such as with trimers and tetramers with 2∶1 and 2∶2 stoichiometry, respectively, as proposed by Breitwieser et al. [68]. At the level of dimer formation, both β_2_AR and IR displayed a higher propensity toward homodimerization than heterodimerization, suggesting homodimers as the basic units engaged in heteromerization. Affinity calculation for trimers and tetramers highlighted differences between β_2_AR and IR. Apparently, neither IR dimers nor trimers could form high affinity interactions with the β_2_AR monomer. However, IR monomers/homodimers can form high affinity heteromers with β_2_AR di−/trimers. Considering that both receptors displayed a higher propensity toward homodimerization and that IR is present in the plasma membrane as a disulfide-linked dimer [28], it is plausible to suggest that high-order 7TMR:RTK oligomers most likely comprise heteromers of homodimers (2∶2 stoichiometry). The computer-assisted peptide scanning survey of the primary structure of β_2_AR and IR revealed the domain encompassing residues 325–364 and 1269–1314 as prospective interaction domains. The identified region is located at the end of the 7^th^ TM domain and C-terminal tail of β_2_AR and almost completely overlaps with helix 8 (helix adjacent to TM7 running along the internal membrane surface) of β_2_AR (residues L324–N357) [69]. A recent study also identified β_2_AR helix 8 as an important dimerization surface region [70]. In addition, this region contains major sites (Tyr350/Tyr354 and Tyr364) for IR-mediated phosphorylation of β_2_AR [3], [71] and consensus sites of Akt-catalyzed phosphorylation (Ser345/Ser 346) [72]. These results are in accordance with previous findings showing that β_2_AR is a substrate for IR and proposing direct interaction between these proteins [3], [71]. The C-terminal cytoplasmic tail–more specifically, the 15-amino acid motif (residues 342–356)–was sufficient to confer the β_1_AR-β_2_AR tail chimera the ability to be regulated by insulin [62].

The prospective interaction domain identified in IR (residues 1269–1314) is positioned in the cytoplasmic part of the IR β chain. This region encompasses the terminal end of the tyrosine kinase catalytic domain and is a part of the C-terminal tail. The C-terminal domain of the IR β subunit has been found to play a key role in the regulation of tyrosine kinase activity [73], [74]. The construct based on proteolytic cleavage used to solve the crystal structure of the IR tyrosine kinase domain of the human IR B-isoform [75] ends at residue 1283, which corresponds to the amino acid at position 1271 in the human IR isoform A used in our study. Therefore, it could be hypothesized that the terminal end of the IR tyrosine kinase domain and a part of the C-terminal tail are involved in the interaction with β_2_AR.

The involvement of intracellular domains (C-terminal tail and ICL3) was found to be fundamental for heterodimerization between the cannabinoid CB_1_ receptor, adenosine A_2A_ and dopamine D_2_ receptors, thus favoring the idea that electrostatic interactions between intracellular domains are more predominant in receptor heteromers and constitute a general mechanism for receptor heteromerization [76].

In summary, BRET data and ISM bioinformatics provided evidence for direct interaction and higher-order β_2_AR:IR oligomer formation that we hypothesize comprise heteromers of homodimers and identified prospective intracellular interaction domains engaged in heteromerization. In this regard, 7TMR:RTK heteromers could potentially generate a basis for the design of new therapeutics that can compete with today’s epidemics, such as type-2 diabetes, obesity and cardiovascular diseases.

## Supporting Information

Figure S1 **HA-β_2_AR internalization as quantified by ELISA.** Cells transiently transfected with HA-β_2_AR (open bars) or HA-β_2_AR together with IR (hatched bars) at a 1∶1 cDNA ratio were incubated at 37°C with either isoproterenol (10 µM), insulin (0.1 µM) or combination of both ligands for 30 min. The amount of internalized receptor was then calculated from the decrease in the level of surface-expressed receptor after ligand treatment compared with untreated, control cells. Data are expressed as the means ± S.E. from three independent experiments performed in triplicate.
(TIF)

Figure S2 **Correlation between total luminescence and fluorescence and the corresponding number of β_2_AR binding sites.** HEK-293 cells were transfected with increasing amounts of HA-β_2_AR-RLuc8 (A) or β_2_AR-GFP^2^ (B) encoding constructs. The β_2_AR receptor density (B_max_) was determined by radioligand binding assays using [^125^I]-iodopindolol as a tracer as described in the Material and methods section. Total luminescence was measured after the addition of the RLuc8 substrate coelenterazine 400a. Total fluorescence was measured with an excitation filter at 380 nm and an emission filter at 515 nm. The linear regression curves were generated using GraphPad Prism 5.0. R^2^ fit values of 0.9705 and 0.9861 were obtained for HA-β_2_AR-RLuc8 (A) and β_2_AR-GFP^2^ (B), respectively.
(TIF)

Figure S3 **Relationship between receptor-RLuc8 and receptor-GFP^2^ constructs expression.** Expression levels of RLuc8- and GFP^2^-tagged constructs used in BRET^2^ saturation assays were also monitored by luminescence and fluorescence measurements. Total luminescence was measured after the addition of the RLuc8 substrate coelenterazine 400a. Total fluorescence was measured with an excitation filter at 380 nm and an emission filter at 515 nm. Data are expressed as the means±S.E. of 3–5 independent saturation experiments.
(TIF)

Figure S4 **Random collisions between the RLuc8-tagged receptors and membrane-inserted GFP^2^-tagged construct (GFP^2^-17aa).** HEK-293 cells were transiently cotransfected with a constant amount of RLuc8-tagged receptors and increasing amounts of GFP^2^-17aa encoding construct. BRET^2^ values were plotted as a function of the ratio between the total fluorescence/total luminescence (GFP^2^/RLuc8 ratio). Total luminescence was measured after the addition of the RLuc8 substrate coelenterazine 400a. Total fluorescence was measured with an excitation filter at 380 nm and an emission filter at 515 nm. Increasing the concentration of GFP^2^-17aa in cells expressing either the IR-RLuc8 or β_2_AR-RLuc8 resulted in high, but nonspecific linear increase of the BRET^2^ signal. Data are expressed as the means ± S.E. from three independent experiments performed in triplicate. Representative BRET^2^ saturation curves of β_2_AR and IR homomers are shown for comparison**.**
(TIF)

Figure S5 **Comparison of theoretical BRET saturation curves with different affinities for trimer formation.** Shown are simulated BRET saturation curves for case with the same affinity for AAD and ADD formation (solid line) and two special cases with different affinities for formation of AAD compared to ADD (hatched and dotted lines). Note that in all three cases the AD_50_ values are different. A: acceptor; D: donor.
(TIF)

Figure S6 **Numerical simulation of heterologous BRET competition assay for trimers**. Comparison of simulated BRET competition curves for trimers with the same (E_1_ = E_2_ = 0.1) and different (E_1_ = 0.1, E_2_ = 0.3) energy transfer ratios for ADD and ADW, where A, D and W are concentrations of acceptor (A = 1), donor (D = 1) and (W) wild type receptors i.e. competitor. Transient increase in BRET signal is observed in the case of different (E_1_ = 0.1, E_2_ = 0.3) energy transfer ratios (dotted line). BRET_0_ is the BRET signal obtained in the absence of competitor.
(TIF)

Figure S7 **Cross-spectrum (CS) of (A) wild type β_2_AR and IR, (B) scrambled β_2_AR and wild type IR and (C) scrambled IR and wild type β_2_AR.** Note that the value of amplitudes at the characteristic peak F(0.216) is higher in CS of two wild type proteins (panel A) compared to the CS of wild type and scrambled proteins (panels B and C).
(TIF)

## References

[1] Barnes PJ (2006) Receptor heterodimerization: a new level of cross-talk. J Clin Invest 116: 1210–1212. 16670762 10.1172/JCI28535 PMC1451216

[2] White MF, Kahn CR (1994) The insulin signaling system. J Biol Chem 269: 1–4. 8276779

[3] Baltensperger K, Karoor V, Paul H, Ruoho A, Czech MP, et al (1996) The β-adrenergic receptor is a substrate for the insulin receptor tyrosine kinase. J Biol Chem 271: 1061–1064. 8557631 10.1074/jbc.271.2.1061

[4] Wang H, Doronin S, Malbon CC (2000) Insulin activation of mitogen-activated protein kinases Erk1,2 is amplified via β-adrenergic receptor expression and requires the integrity of the Tyr350 of the receptor. J Biol Chem 275: 36086–36093. 10940302 10.1074/jbc.M004404200

[5] Shih M, Malbon CC (1998) Serum and insulin induce a Grb2-dependent shift in agonist affinity of β-adrenergic receptors. Cell Signal 10: 575–582. 9794256 10.1016/s0898-6568(97)00195-2

[6] Shumay E, Gavi S, Wang HY, Malbon CC (2004) Trafficking of β_2_-adrenergic receptors: insulin and β-agonists regulate internalization by distinct cytoskeletal pathways. J Cell Sci 117: 593–600. 14709719 10.1242/jcs.00890

[7] Fu Q, Xu B, Liu Y, Parikh D, Li J, et al (2014) Insulin inhibits cardiac contractility by inducing a G_i_-biased β_2_ adrenergic signaling in hearts. Diabetes 63: 2676–2689. 24677713 10.2337/db13-1763 PMC4113065

[8] Heni M, Hennige AM, Peter A, Siegel-Axel D, Ordelheide AM, et al (2011) Insulin promotes glycogen storage and cell proliferation in primary human astrocytes. PLoS One 6: e21594. 21738722 10.1371/journal.pone.0021594 PMC3124526

[9] Lange LA, Norris JM, Langefeld CD, Nicklas BJ, Wagenknecht LE, et al (2005) Association of adipose tissue deposition and beta-2 adrenergic receptor variants: the IRAS family study. Int J Obes (Lond) 29: 449–457. 15672110 10.1038/sj.ijo.0802883

[10] Liggett SB, Shah SD, Cryer PE (1988) Characterization of beta-adrenergic receptors of human skeletal muscle obtained by needle biopsy. Am J Physiol 254: E795–E798. 2837096 10.1152/ajpendo.1988.254.6.E795

[11] Angers S, Salahpour A, Bouvier M (2002) Dimerization: an emerging concept for G protein-coupled receptor ontogeny and function. Annu Rev Pharmacol Toxicol 42: 409–435. 11807178 10.1146/annurev.pharmtox.42.091701.082314

[12] Lee SP, O’Dowd BF, George SR (2003) Homo- and hetero-oligomerization of G protein-coupled receptors. Life Sci 74: 173–180. 14607244 10.1016/j.lfs.2003.09.028

[13] Achour L, Kamal M, Jockers R, Marullo S (2011) Using quantitative BRET to assess G protein-coupled receptor homo- and heterodimerization. Methods Mol Biol 756: 183–200. 21870226 10.1007/978-1-61779-160-4_9

[14] Ayoub MA, Pfleger KD (2010) Recent advances in bioluminescence resonance energy transfer technologies to study GPCR heteromerization. Curr Opin Pharmacol 10: 44–52. 19897419 10.1016/j.coph.2009.09.012

[15] Ferre S, Baler R, Bouvier M, Caron MG, Devi LA, et al (2009) Building a new conceptual framework for receptor heteromers. Nat Chem Biol 5: 131–134. 19219011 10.1038/nchembio0309-131 PMC2681085

[16] Ferre S, Franco R (2010) Oligomerization of G-protein-coupled receptors: a reality. Curr Opin Pharmacol 10: 1–5. 20015687 10.1016/j.coph.2009.11.002 PMC2825689

[17] Gurevich VV, Gurevich EV (2008) GPCR monomers and oligomers: it takes all kinds. Trends Neurosci 31: 74–81. 18199492 10.1016/j.tins.2007.11.007 PMC2366802

[18] Palczewski K (2010) Oligomeric forms of G protein-coupled receptors (GPCRs). Trends Biochem Sci 35: 595–600. 20538466 10.1016/j.tibs.2010.05.002 PMC2937196

[19] Angers S, Salahpour A, Joly E, Hilairet S, Chelsky D, et al (2000) Detection of β_2_-adrenergic receptor dimerization in living cells using bioluminescence resonance energy transfer (BRET). Proc Natl Acad Sci U S A 97: 3684–3689. 10725388 10.1073/pnas.060590697 PMC16300

[20] Breit A, Lagace M, Bouvier M (2004) Hetero-oligomerization between β_2_- and β_3_-adrenergic receptors generates a β-adrenergic signaling unit with distinct functional properties. J Biol Chem 279: 28756–28765. 15123695 10.1074/jbc.M313310200

[21] Lavoie C, Mercier JF, Salahpour A, Umapathy D, Breit A, et al (2002) β_1_/β_2_-adrenergic receptor heterodimerization regulates β_2_-adrenergic receptor internalization and ERK signaling efficacy. J Biol Chem 277: 35402–35410. 12140284 10.1074/jbc.M204163200

[22] McGraw DW, Mihlbachler KA, Schwarb MR, Rahman FF, Small KM, et al (2006) Airway smooth muscle prostaglandin-EP1 receptors directly modulate β_2_-adrenergic receptors within a unique heterodimeric complex. J Clin Invest 116: 1400–1409. 16670773 10.1172/JCI25840 PMC1451203

[23] Mercier JF, Salahpour A, Angers S, Breit A, Bouvier M (2002) Quantitative assessment of β_1_- and β_2_-adrenergic receptor homo- and heterodimerization by bioluminescence resonance energy transfer. J Biol Chem 277: 44925–44931. 12244098 10.1074/jbc.M205767200

[24] Vrecl M, Drinovec L, Elling C, Heding A (2006) Opsin oligomerization in a heterologous cell system. J Recept Signal Transduct Res 26: 505–526. 17118796 10.1080/10799890600932253

[25] Wrzal PK, Devost D, Petrin D, Goupil E, Iorio-Morin C, et al (2012) Allosteric interactions between the oxytocin receptor and the β_2_-adrenergic receptor in the modulation of ERK1/2 activation are mediated by heterodimerization. Cell Signal 24: 342–350. 21963428 10.1016/j.cellsig.2011.09.020

[26] Issad T, Blanquart C, Gonzalez-Yanes C (2007) The use of bioluminescence resonance energy transfer for the study of therapeutic targets: application to tyrosine kinase receptors. Expert Opin Ther Targets 11: 541–556. 17373883 10.1517/14728222.11.4.541

[27] Siddiqui S, Cong WN, Daimon CM, Martin B, Maudsley S (2013) BRET Biosensor Analysis of Receptor Tyrosine Kinase Functionality. Front Endocrinol (Lausanne) 4: 46. 23577003 10.3389/fendo.2013.00046 PMC3620488

[28] De Meyts P (2008) The insulin receptor: a prototype for dimeric, allosteric membrane receptors? Trends Biochem Sci 33: 376–384. 18640841 10.1016/j.tibs.2008.06.003

[29] Boute N, Pernet K, Issad T (2001) Monitoring the activation state of the insulin receptor using bioluminescence resonance energy transfer. Mol Pharmacol 60: 640–645. 11562424

[30] Issad T, Boute N, Pernet K (2002) The activity of the insulin receptor assessed by bioluminescence resonance energy transfer. Ann N Y Acad Sci 973: 120–123. 12485847 10.1111/j.1749-6632.2002.tb04619.x

[31] Issad T, Boute N, Pernet K (2002) A homogenous assay to monitor the activity of the insulin receptor using Bioluminescence Resonance Energy Transfer. Biochem Pharmacol 64: 813–817. 12213574 10.1016/s0006-2952(02)01143-7

[32] Boute N, Boubekeur S, Lacasa D, Issad T (2003) Dynamics of the interaction between the insulin receptor and protein tyrosine-phosphatase 1B in living cells. EMBO Rep 4: 313–319. 12634852 10.1038/sj.embor.embor767 PMC1315895

[33] Nouaille S, Blanquart C, Zilberfarb V, Boute N, Perdereau D, et al (2006) Interaction between the insulin receptor and Grb14: a dynamic study in living cells using BRET. Biochem Pharmacol 72: 1355–1366. 16934761 10.1016/j.bcp.2006.07.018

[34] Kulahin N, Sanni SJ, Slaaby R, Nohr J, Gammeltoft S, et al (2012) A BRET assay for monitoring insulin receptor interactions and ligand pharmacology. J Recept Signal Transduct Res 32: 57–64. 22272819 10.3109/10799893.2011.647351

[35] Kim JG, Kang MJ, Yoon YK, Kim HP, Park J, et al (2012) Heterodimerization of glycosylated insulin-like growth factor-1 receptors and insulin receptors in cancer cells sensitive to anti-IGF1R antibody. PLoS One 7: e33322. 22438913 10.1371/journal.pone.0033322 PMC3306383

[36] Flajolet M, Wang Z, Futter M, Shen W, Nuangchamnong N, et al (2008) FGF acts as a co-transmitter through adenosine A2A receptor to regulate synaptic plasticity. Nat Neurosci 11: 1402–1409. 18953346 10.1038/nn.2216 PMC2779562

[37] Maudsley S, Pierce KL, Zamah AM, Miller WE, Ahn S, et al (2000) The β-adrenergic receptor mediates extracellular signal-regulated kinase activation via assembly of a multi-receptor complex with the epidermal growth factor receptor. J Biol Chem 275: 9572–9580. 10734107 10.1074/jbc.275.13.9572

[38] Waters CM, Connell MC, Pyne S, Pyne NJ (2005) c-Src is involved in regulating signal transmission from PDGFβ receptor-GPCR(s) complexes in mammalian cells. Cell Signal 17: 263–277. 15494217 10.1016/j.cellsig.2004.07.011

[39] Wang C, Buck DC, Yang R, Macey TA, Neve KA (2005) Dopamine D2 receptor stimulation of mitogen-activated protein kinases mediated by cell type-dependent transactivation of receptor tyrosine kinases. J Neurochem 93: 899–909. 15857393 10.1111/j.1471-4159.2005.03055.x

[40] Borroto-Escuela DO, Tarakanov AO, Guidolin D, Ciruela F, Agnati LF, et al (2011) Moonlighting characteristics of G protein-coupled receptors: focus on receptor heteromers and relevance for neurodegeneration. IUBMB Life 63: 463–472. 21698749 10.1002/iub.473

[41] Veljkovic V, Cosic I (1987) A novel method of protein analysis for prediction of biological function: application to tumor toxins. Cancer Biochem Biophys 9: 139–148. 3497707

[42] Veljkovic V, Cosic I, Dimitrijevic B, Lalovic D (1985) Is it possible to analyze DNA and protein sequences by the methods of digital signal processing? IEEE Trans Biomed Eng 32: 337–341. 10.1109/TBME.1985.325549 2581884

[43] Doliana R, Veljkovic V, Prljic J, Veljkovic N, De Lorenzo E, et al (2008) EMILINs interact with anthrax protective antigen and inhibit toxin action in vitro. Matrix Biol 27: 96–106. 17988845 10.1016/j.matbio.2007.09.008

[44] Mancini M, Veljkovic N, Leo E, Aluigi M, Borsi E, et al (2012) Cytoplasmatic compartmentalization by Bcr-Abl promotes TET2 loss-of-function in chronic myeloid leukemia. J Cell Biochem 113: 2765–2774. 22467095 10.1002/jcb.24154

[45] Svendsen AM, Vrecl M, Ellis TM, Heding A, Kristensen JB, et al (2008) Cooperative binding of insulin-like Peptide 3 to a dimeric relaxin family peptide receptor 2. Endocrinology 149: 1113–1120. 18063691 10.1210/en.2007-0412

[46] Vrecl M, Norregaard PK, Almholt DL, Elster L, Pogacnik A, et al (2009) β-arrestin-based BRET^2^ screening assay for the “non”-β-arrestin binding CB1 receptor. J Biomol Screen 14: 371–380. 19403920 10.1177/1087057109333101

[47] Vrecl M, Jorgensen R, Pogacnik A, Heding A (2004) Development of a BRET^2^ screening assay using β-arrestin 2 mutants. J Biomol Screen 9: 322–333. 15191649 10.1177/1087057104263212

[48] Ramsay D, Kellett E, McVey M, Rees S, Milligan G (2002) Homo- and hetero-oligomeric interactions between G-protein-coupled receptors in living cells monitored by two variants of bioluminescence resonance energy transfer (BRET): hetero-oligomers between receptor subtypes form more efficiently than between less closely related sequences. Biochem J 365: 429–440. 11971762 10.1042/BJ20020251 PMC1222697

[49] Kubale V, Abramovic Z, Pogacnik A, Heding A, Sentjurc M, et al (2007) Evidence for a role of caveolin-1 in neurokinin-1 receptor plasma-membrane localization, efficient signaling, and interaction withβ-arrestin 2. Cell Tissue Res 330: 231–245. 17713785 10.1007/s00441-007-0462-y

[50] Veatch W, Stryer L (1977) The dimeric nature of the gramicidin A transmembrane channel: conductance and fluorescence energy transfer studies of hybrid channels. Journal of molecular biology 113: 89–102. 69713 10.1016/0022-2836(77)90042-0

[51] Veljkovic N, Glisic S, Prljic J, Perovic V, Botta M, et al (2008) Discovery of new therapeutic targets by the informational spectrum method. Curr Protein Pept Sci 9: 493–506. 18855700 10.2174/138920308785915245

[52] Veljkovic V, Niman HL, Glisic S, Veljkovic N, Perovic V, et al (2009) Identification of hemagglutinin structural domain and polymorphisms which may modulate swine H1N1 interactions with human receptor. BMC Struct Biol 9: 62. 19785758 10.1186/1472-6807-9-62 PMC2760557

[53] Veljkovic V, Veljkovic N, Muller CP, Muller S, Glisic S, et al (2009) Characterization of conserved properties of hemagglutinin of H5N1 and human influenza viruses: possible consequences for therapy and infection control. BMC Struct Biol 9: 21. 19351406 10.1186/1472-6807-9-21 PMC2679750

[54] Drinovec L, Kubale V, Nohr Larsen J, Vrecl M (2012) Mathematical models for quantitative assessment of bioluminescence resonance energy transfer: application to seven transmembrane receptors oligomerization. Front Endocrinol (Lausanne) 3: 104. 22973259 10.3389/fendo.2012.00104 PMC3428587

[55] Calebiro D, Rieken F, Wagner J, Sungkaworn T, Zabel U, et al (2013) Single-molecule analysis of fluorescently labeled G-protein-coupled receptors reveals complexes with distinct dynamics and organization. Proc Natl Acad Sci U S A 110: 743–748. 23267088 10.1073/pnas.1205798110 PMC3545784

[56] Ferre S, Casado V, Devi LA, Filizola M, Jockers R, et al (2014) G Protein-Coupled Receptor Oligomerization Revisited: Functional and Pharmacological Perspectives Pharmacol Rev. 66: 413–434. 10.1124/pr.113.008052 PMC3973609 24515647

[57] Scarselli M, Donaldson JG (2009) Constitutive internalization of G protein-coupled receptors and G proteins via clathrin-independent endocytosis. J Biol Chem 284: 3577–3585. 19033440 10.1074/jbc.M806819200 PMC2635037

[58] Oakley RH, Laporte SA, Holt JA, Barak LS, Caron MG (1999) Association of β-arrestin with G protein-coupled receptors during clathrin-mediated endocytosis dictates the profile of receptor resensitization. J Biol Chem 274: 32248–32257. 10542263 10.1074/jbc.274.45.32248

[59] Seachrist JL, Anborgh PH, Ferguson SS (2000) β_2_-adrenergic receptor internalization, endosomal sorting, and plasma membrane recycling are regulated by rab GTPases. J Biol Chem 275: 27221–27228. 10854436 10.1074/jbc.M003657200

[60] Di Certo MG, Batassa EM, Casella I, Serafino A, Floridi A, et al (2008) Delayed internalization and lack of recycling in a beta_2_-adrenergic receptor fused to the G protein alpha-subunit. BMC Cell Biol 9: 56. 18840275 10.1186/1471-2121-9-56 PMC2569931

[61] Karoor V, Wang L, Wang HY, Malbon CC (1998) Insulin stimulates sequestration of β-adrenergic receptors and enhanced association of β-adrenergic receptors with Grb2 via tyrosine 350. J Biol Chem 273: 33035–33041. 9830057 10.1074/jbc.273.49.33035

[62] Gavi S, Yin D, Shumay E, Wang HY, Malbon CC (2005) The 15-amino acid motif of the C terminus of the β_2_-adrenergic receptor is sufficient to confer insulin-stimulated counterregulation to the β_1_-adrenergic receptor. Endocrinology 146: 450–457. 15388645 10.1210/en.2004-0595

[63] Dacres H, Michie M, Wang J, Pfleger KD, Trowell SC (2012) Effect of enhanced Renilla luciferase and fluorescent protein variants on the Forster distance of Bioluminescence resonance energy transfer (BRET). Biochem Biophys Res Commun 425: 625–629. 22877756 10.1016/j.bbrc.2012.07.133

[64] Mondal S, Johnston JM, Wang H, Khelashvili G, Filizola M, et al (2013) Membrane driven spatial organization of GPCRs. Sci Rep 3: 2909. 24105260 10.1038/srep02909 PMC3793225

[65] Ayoub MA, Levoye A, Delagrange P, Jockers R (2004) Preferential formation of MT_1_/MT_2_ melatonin receptor heterodimers with distinct ligand interaction properties compared with MT_2_ homodimers. Mol Pharmacol 66: 312–321. 15266022 10.1124/mol.104.000398

[66] Terrillon S, Durroux T, Mouillac B, Breit A, Ayoub MA, et al (2003) Oxytocin and vasopressin V1a and V2 receptors form constitutive homo- and heterodimers during biosynthesis. Mol Endocrinol 17: 677–691. 12554793 10.1210/me.2002-0222

[67] Goin JC, Nathanson NM (2006) Quantitative analysis of muscarinic acetylcholine receptor homo- and heterodimerization in live cells: regulation of receptor down-regulation by heterodimerization. J Biol Chem 281: 5416–5425. 16368694 10.1074/jbc.M507476200

[68] Breitwieser GE (2004) G protein-coupled receptor oligomerization: implications for G protein activation and cell signaling. Circ Res 94: 17–27. 14715532 10.1161/01.RES.0000110420.68526.19

[69] Katragadda M, Maciejewski MW, Yeagle PL (2004) Structural studies of the putative helix 8 in the human β_2_ adrenergic receptor: an NMR study. Biochim Biophys Acta 1663: 74–81. 15157609 10.1016/j.bbamem.2004.01.012

[70] Ghosh A, Sonavane U, Joshi R (2014) Multiscale modelling to understand the self-assembly mechanism of human β2-adrenergic receptor in lipid bilayer. Comput Biol Chem 48: 29–39. 24291490 10.1016/j.compbiolchem.2013.11.002

[71] Doronin S, Wang Hy HY, Malbon CC (2002) Insulin stimulates phosphorylation of the β_2_-adrenergic receptor by the insulin receptor, creating a potent feedback inhibitor of its tyrosine kinase. J Biol Chem 277: 10698–10703. 11782469 10.1074/jbc.M109432200

[72] Doronin S, Shumay E, Wang HY, Malbon CC (2002) Akt mediates sequestration of the β_2_-adrenergic receptor in response to insulin. J Biol Chem 277: 15124–15131. 11809767 10.1074/jbc.M108771200

[73] Mothe I, Tartare S, Kowalski-Chauvel A, Kaliman P, Van Obberghen E, et al (1995) Tyrosine kinase activity of a chimeric insulin-like-growth-factor-1 receptor containing the insulin receptor C-terminal domain. Comparison with the tyrosine kinase activities of the insulin and insulin-like-growth-factor-1 receptors using a cell-free system. Eur J Biochem 228: 842–848. 7737184 10.1111/j.1432-1033.1995.0842m.x

[74] Soni P, Lakkis M, Poy MN, Fernstrom MA, Najjar SM (2000) The differential effects of pp120 (Ceacam 1) on the mitogenic action of insulin and insulin-like growth factor 1 are regulated by the nonconserved tyrosine 1316 in the insulin receptor. Mol Cell Biol 20: 3896–3905. 10805733 10.1128/mcb.20.11.3896-3905.2000 PMC85733

[75] Hubbard SR, Wei L, Ellis L, Hendrickson WA (1994) Crystal structure of the tyrosine kinase domain of the human insulin receptor. Nature 372: 746–754. 7997262 10.1038/372746a0

[76] Navarro G, Ferre S, Cordomi A, Moreno E, Mallol J, et al (2010) Interactions between intracellular domains as key determinants of the quaternary structure and function of receptor heteromers. J Biol Chem 285: 27346–27359. 20562103 10.1074/jbc.M110.115634 PMC2930733

