# Supplementary figures and images for: Demonstration of a Direct Interaction between β2-Adrenergic Receptor and Insulin Receptor by BRET and Bioinformatics
Source: PLoS One. 2014 Nov 17;9(11):e112664. doi: 10.1371/journal.pone.0112664 (PMC4234468; doi:10.1371/journal.pone.0112664)

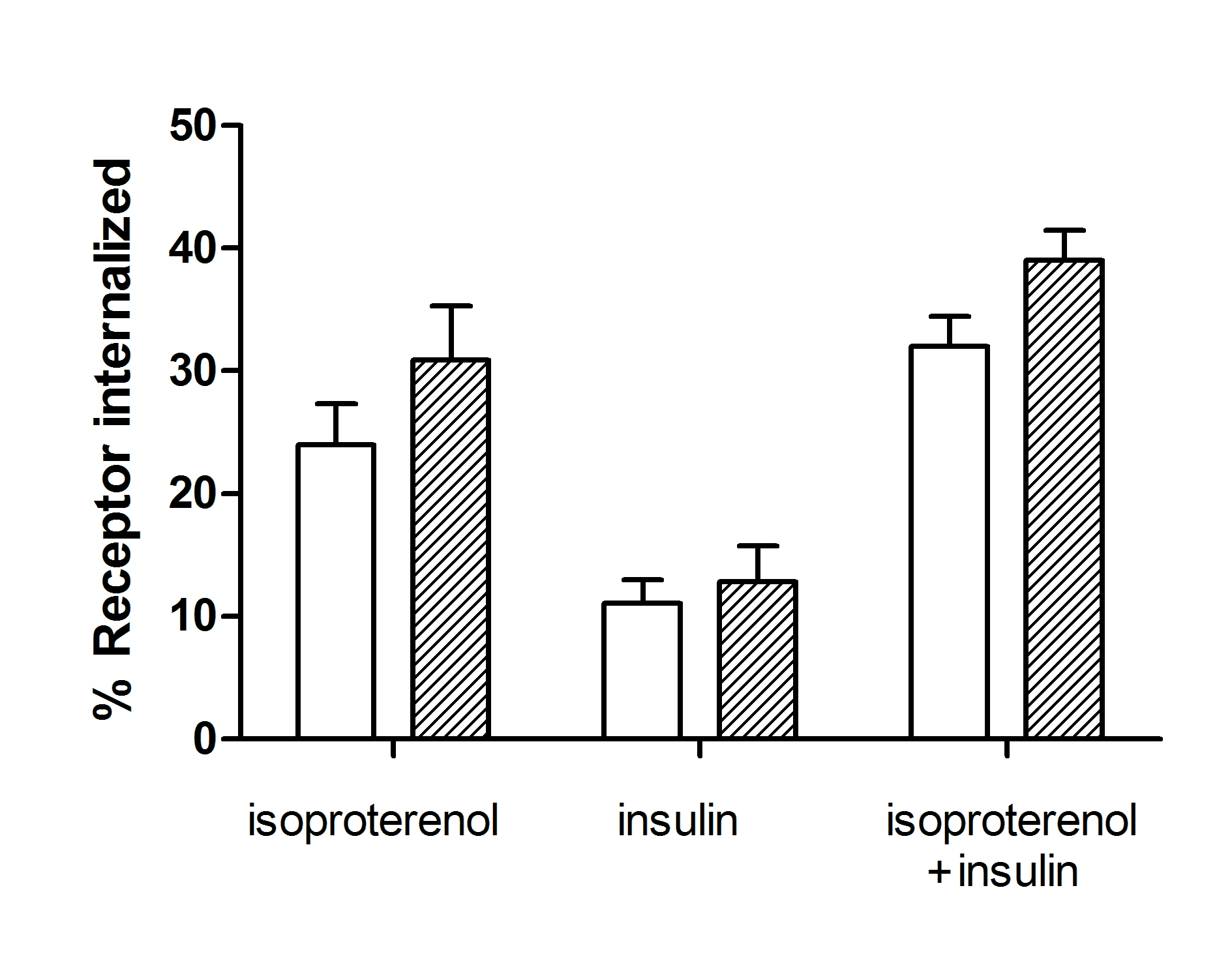

Supplement: Figure S1 — HA-β2AR internalization as quantified by ELISA. Cells transiently transfected with HA-β2AR (open bars) or HA-β2AR together with IR (hatched bars) at a 1∶1 cDNA ratio were incubated at 37°C with either isoproterenol (10 µM), insulin (0.1 µM) or combination of both ligands for 30 min. The amount of internalized receptor was then calculated from the decrease in the level of surface-expressed receptor after ligand treatment compared with untreated, control cells. Data are expressed as the means ± S.E. from three independent experiments performed in triplicate. (TIF) [file pone.0112664.s001.tif]

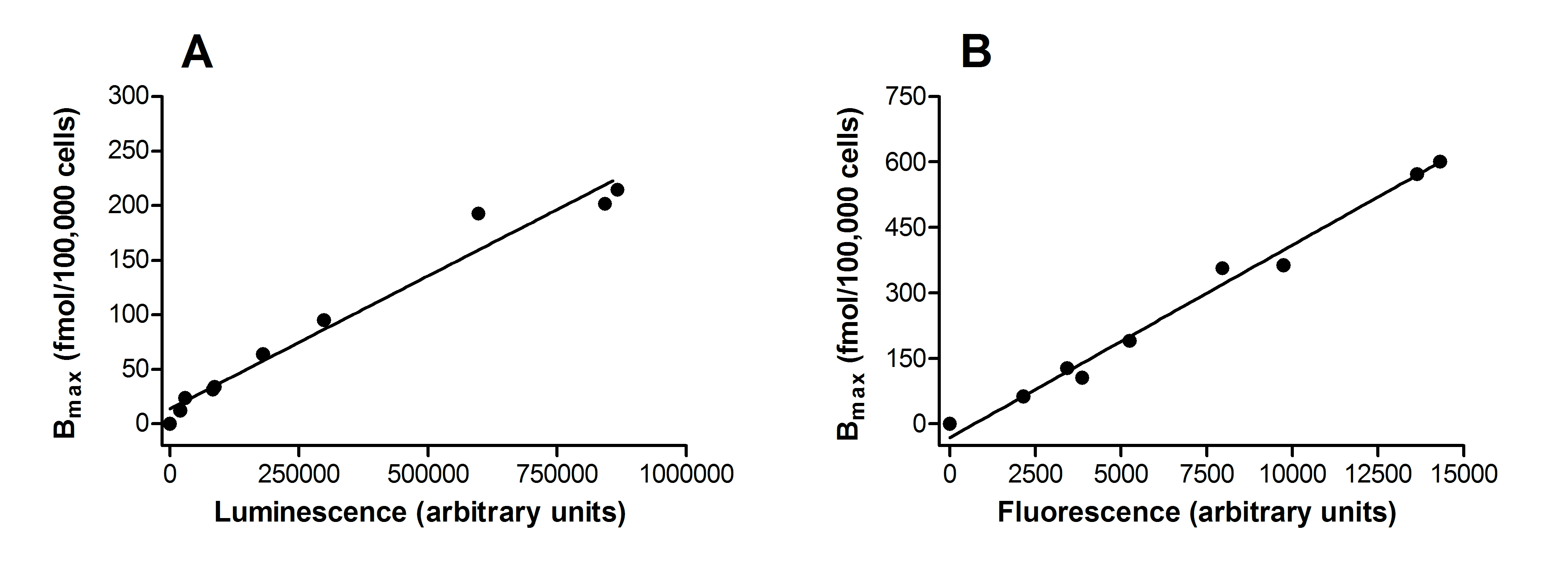

Supplement: Figure S2 — Correlation between total luminescence and fluorescence and the corresponding number of β2AR binding sites. HEK-293 cells were transfected with increasing amounts of HA-β2AR-RLuc8 (A) or β2AR-GFP2 (B) encoding constructs. The β2AR receptor density (Bmax) was determined by radioligand binding assays using [125I]-iodopindolol as a tracer as described in the Material and methods section. Total luminescence was measured after the addition of the RLuc8 substrate coelenterazine 400a. Total fluorescence was measured with an excitation filter at 380 nm and an emission filter at 515 nm. The linear regression curves were generated using GraphPad Prism 5.0. R2 fit values of 0.9705 and 0.9861 were obtained for HA-β2AR-RLuc8 (A) and β2AR-GFP2 (B), respectively. (TIF) [file pone.0112664.s002.tif]

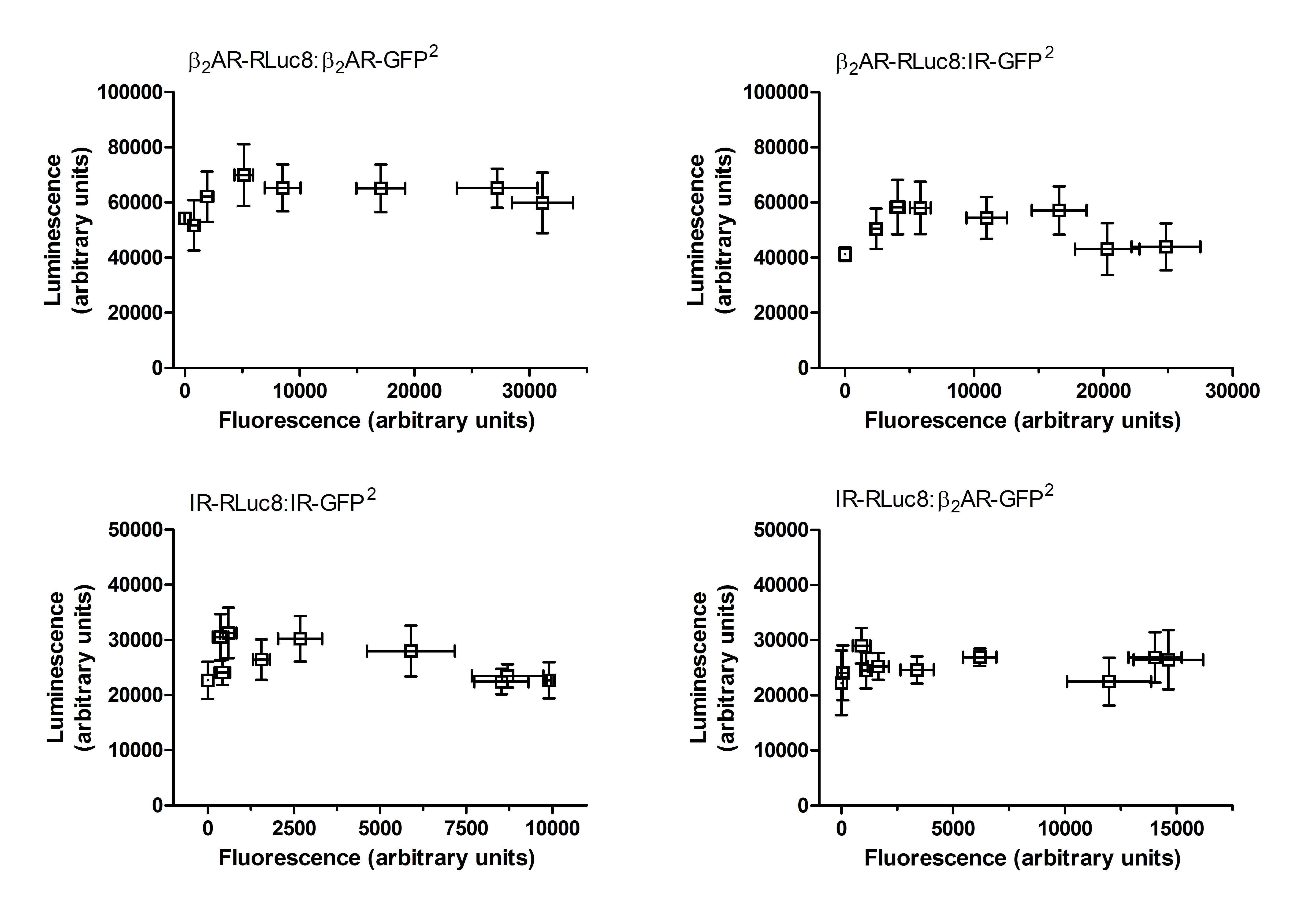

Supplement: Figure S3 — Relationship between receptor-RLuc8 and receptor-GFP2 constructs expression. Expression levels of RLuc8- and GFP2-tagged constructs used in BRET2 saturation assays were also monitored by luminescence and fluorescence measurements. Total luminescence was measured after the addition of the RLuc8 substrate coelenterazine 400a. Total fluorescence was measured with an excitation filter at 380 nm and an emission filter at 515 nm. Data are expressed as the means±S.E. of 3–5 independent saturation experiments. (TIF) [file pone.0112664.s003.tif]

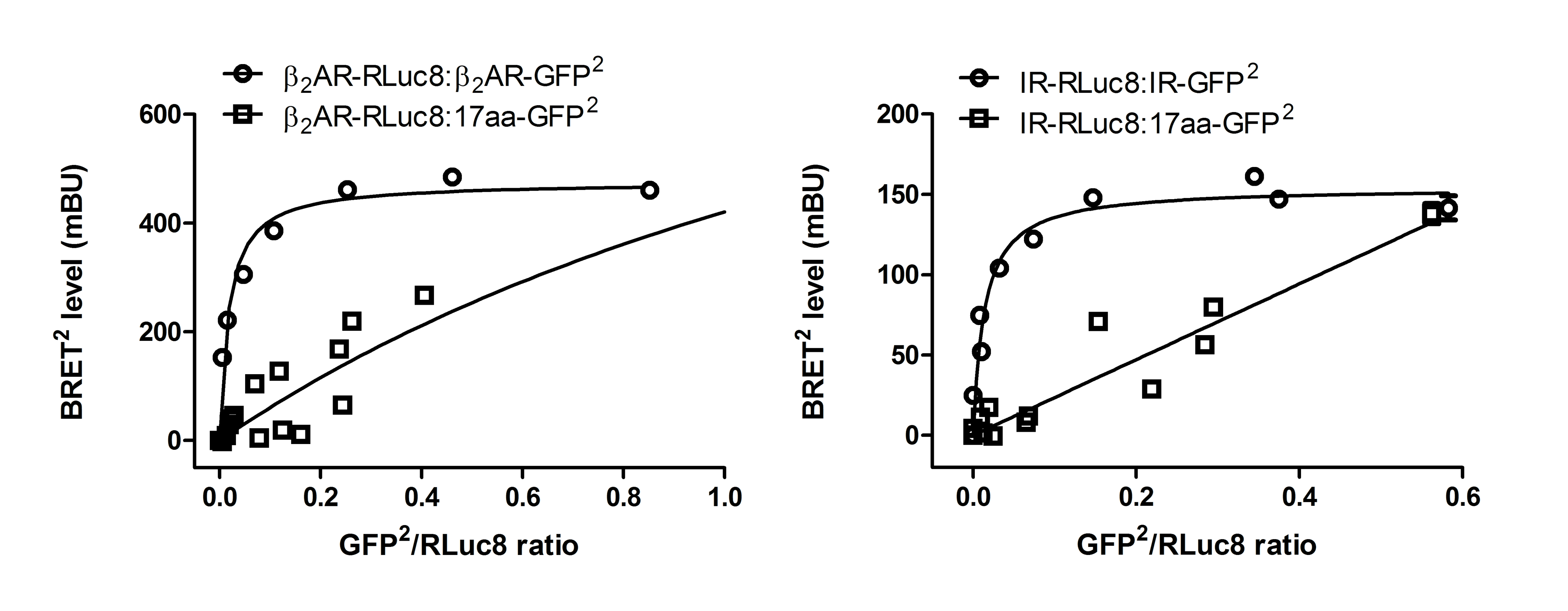

Supplement: Figure S4 — Random collisions between the RLuc8-tagged receptors and membrane-inserted GFP2-tagged construct (GFP2-17aa). HEK-293 cells were transiently cotransfected with a constant amount of RLuc8-tagged receptors and increasing amounts of GFP2-17aa encoding construct. BRET2 values were plotted as a function of the ratio between the total fluorescence/total luminescence (GFP2/RLuc8 ratio). Total luminescence was measured after the addition of the RLuc8 substrate coelenterazine 400a. Total fluorescence was measured with an excitation filter at 380 nm and an emission filter at 515 nm. Increasing the concentration of GFP2-17aa in cells expressing either the IR-RLuc8 or β2AR-RLuc8 resulted in high, but nonspecific linear increase of the BRET2 signal. Data are expressed as the means ± S.E. from three independent experiments performed in triplicate. Representative BRET2 saturation curves of β2AR and IR homomers are shown for comparison. (TIF) [file pone.0112664.s004.tif]

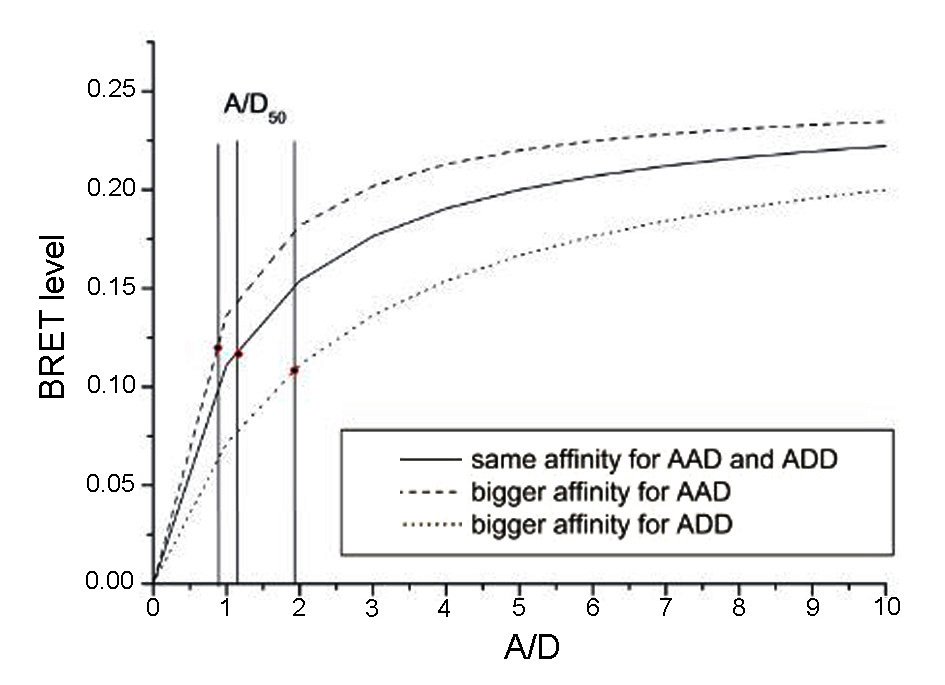

Supplement: Figure S5 — Comparison of theoretical BRET saturation curves with different affinities for trimer formation. Shown are simulated BRET saturation curves for case with the same affinity for AAD and ADD formation (solid line) and two special cases with different affinities for formation of AAD compared to ADD (hatched and dotted lines). Note that in all three cases the AD50 values are different. A: acceptor; D: donor. (TIF) [file pone.0112664.s005.tif]

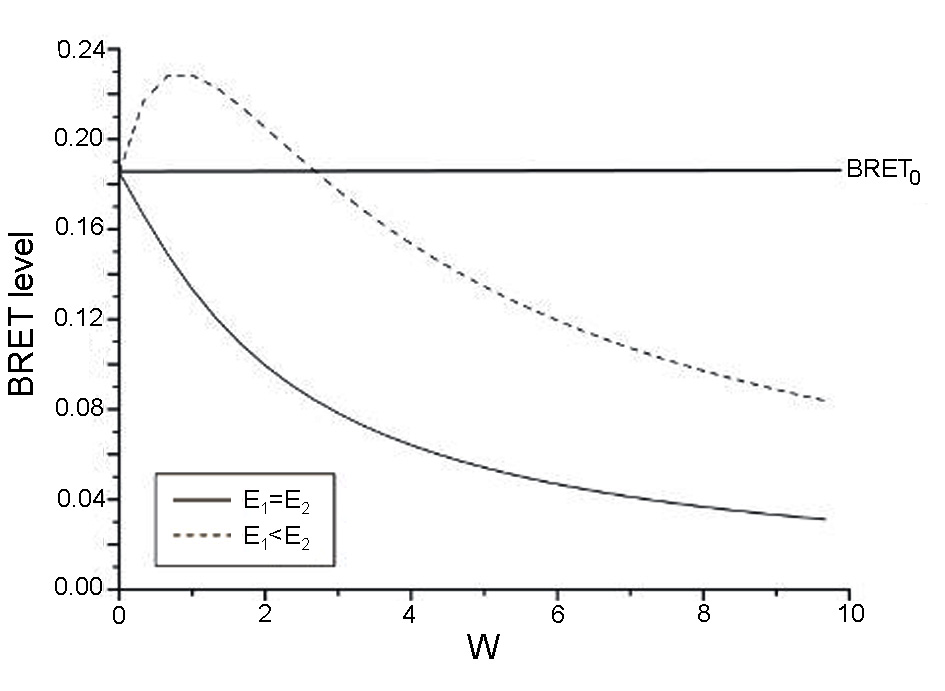

Supplement: Figure S6 — Numerical simulation of heterologous BRET competition assay for trimers. Comparison of simulated BRET competition curves for trimers with the same (E1 = E2 = 0.1) and different (E1 = 0.1, E2 = 0.3) energy transfer ratios for ADD and ADW, where A, D and W are concentrations of acceptor (A = 1), donor (D = 1) and (W) wild type receptors i.e. competitor. Transient increase in BRET signal is observed in the case of different (E1 = 0.1, E2 = 0.3) energy transfer ratios (dotted line). BRET0 is the BRET signal obtained in the absence of competitor. (TIF) [file pone.0112664.s006.tif]

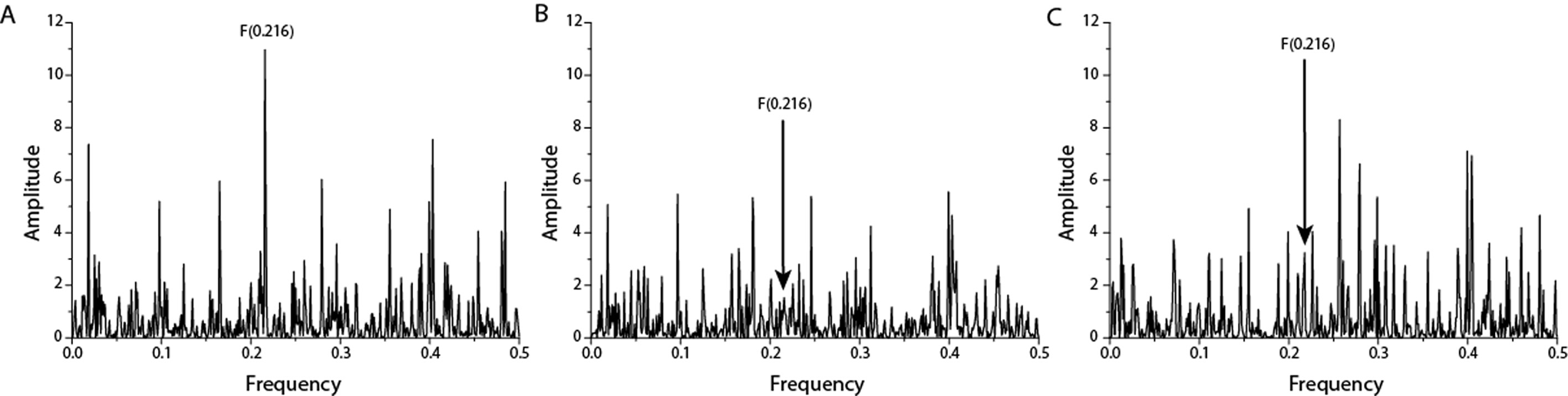

Supplement: Figure S7 — Cross-spectrum (CS) of (A) wild type β2AR and IR, (B) scrambled β2AR and wild type IR and (C) scrambled IR and wild type β2AR. Note that the value of amplitudes at the characteristic peak F(0.216) is higher in CS of two wild type proteins (panel A) compared to the CS of wild type and scrambled proteins (panels B and C). (TIF) [file pone.0112664.s007.tif]
